# Clonal tracing reveals diverse patterns of response to immune checkpoint blockade

**DOI:** 10.1186/s13059-020-02166-1

**Published:** 2020-10-15

**Authors:** Shengqing Stan Gu, Xiaoqing Wang, Xihao Hu, Peng Jiang, Ziyi Li, Nicole Traugh, Xia Bu, Qin Tang, Chenfei Wang, Zexian Zeng, Jingxin Fu, Cliff Meyer, Yi Zhang, Paloma Cejas, Klothilda Lim, Jin Wang, Wubing Zhang, Collin Tokheim, Avinash Das Sahu, Xiaofang Xing, Benjamin Kroger, Zhangyi Ouyang, Henry Long, Gordon J. Freeman, Myles Brown, X. Shirley Liu

**Affiliations:** 1 Department of Data Science, Dana-Farber Cancer Institute, Boston, MA 02215 USA; 2 Department of Biostatistics, Harvard T.H. Chan School of Public Health, Boston, MA 02215 USA; 3 Center for Functional Cancer Epigenetics, Dana-Farber Cancer Institute, Boston, MA 02215 USA; 4 Department of Medical Oncology, Dana-Farber Cancer Institute, Boston, MA 02215 USA; 5 Harvard Medical School, Boston, MA 02115 USA; 6 Clinical Translational Research Center, Shanghai Pulmonary Hospital, School of Life Science and Technology, Tongji University, Shanghai, 200433 China; 7 Division of Gastrointestinal Cancer Translational Research Laboratory, Peking University Cancer Hospital & Institute, Beijing, 100142 China; 8 University of Texas Southwestern Medical School, Dallas, TX 75390 USA

**Keywords:** Clonal tracing, Immune checkpoint blockade, Heterogeneity, Tumor microenvironment, Mathematical modeling

## Abstract

**Background:**

Immune checkpoint blockade (ICB) therapy has improved patient survival in a variety of cancers, but only a minority of cancer patients respond. Multiple studies have sought to identify general biomarkers of ICB response, but elucidating the molecular and cellular drivers of resistance for individual tumors remains challenging. We sought to determine whether a tumor with defined genetic background exhibits a stereotypic or heterogeneous response to ICB treatment.

**Results:**

We establish a unique mouse system that utilizes clonal tracing and mathematical modeling to monitor the growth of each cancer clone, as well as the bulk tumor, in response to ICB. We find that tumors derived from the same clonal populations showed heterogeneous ICB response and diverse response patterns. Primary response is associated with higher immune infiltration and leads to enrichment of pre-existing ICB-resistant cancer clones. We further identify several cancer cell-intrinsic gene expression signatures associated with ICB resistance, including increased interferon response genes and glucocorticoid response genes. These findings are supported by clinical data from ICB treatment cohorts.

**Conclusions:**

Our study demonstrates diverse response patterns from the same ancestor cancer cells in response to ICB. This suggests the value of monitoring clonal constitution and tumor microenvironment over time to optimize ICB response and to design new combination therapies. Furthermore, as ICB response may enrich for cancer cell-intrinsic resistance signatures, this can affect interpretations of tumor RNA-seq data for response-signature association studies.

## Background

Immune checkpoint blockade (ICB) therapy, primarily anti-PD-1/PD-L1 and anti-CTLA-4, has been shown to induce remarkable response across different cancers in multiple clinical cohorts [1–8]. However, only a minority of patients respond to ICB, and acquired resistance can occur in some early responders [9]. The mechanisms of resistance have been of extensive interest. On the one hand, multiple cancer cell-intrinsic mechanisms have been identified, including lack of a high neoantigen load [2, 3, 10, 11], impaired response or prolonged exposure to IFNγ [12–15], defects in antigen presentation [16, 17], activation of certain oncogenic pathways/signatures [18–20], and other mechanisms [20, 21]. On the other hand, multiple cancer cell-extrinsic mechanisms have been reported to modulate ICB response, involving microenvironmental T cells [22–27], B cells [28–30], myeloid cells [31–33], natural killer cells [34], cancer-associated fibroblasts [34, 35], and gut microbiota [36–38]. The simultaneous contributions of both cancer cell-intrinsic and cancer cell-extrinsic factors present a challenge in prediction of ICB response for each tumor.

As the major mechanism of action for ICB is thought to be through the stimulation of T cell cytotoxicity in the tumors, two distinct models of tumor immune evasion have been proposed: the induction of T cell dysfunction in tumors with high infiltration of cytotoxic T lymphocytes (CTLs) and the suppression of T cell infiltration in tumors with low CTL levels [39, 40]. Our group previously developed a computational method, TIDE, which comprehensively evaluates T cell dysfunction and T cell exclusion signatures from the pre-treatment tumor gene expression data to predict patient responses to ICB treatment [41]. TIDE demonstrated promising performance in predicting patient responses based on published ICB clinical trials and also revealed new candidate ICB-resistance regulators. However, the performance of TIDE still varies across different clinical cohorts.

Tumors of different tissue origins and genetic backgrounds have different prognoses in response to ICB [9, 42], suggesting that different gene expression and/or mutation profiles can have a strong influence on ICB response. However, it remains unclear whether tumors with a defined genetic background have a stereotypic or heterogeneous response to ICB. Answering this question is largely limited by a lack of experimental models. No two naturally occurring tumors are identical, making it difficult to study this question directly in human tumors. Patient-derived xenograft (PDX) models suffer from a lack of adaptive immune system in the recipient mice, precluding the assessment of ICB response. Genetically engineered mouse (GEM) models offer an intact immune system, but usually lack a sufficient mutational burden to elicit an effective immune response. Several syngeneic transplantation models harbor sufficient mutation loads and show response to ICB treatment in vivo, providing a powerful tool to study this question. However, thus far, no studies have assessed the heterogeneity of clonal evolution in response to ICB.

Clonal tracing is a powerful technique that can track and quantify the abundance of different clonal lineages with remarkable sensitivity and reproducibility. It has been used to interrogate a variety of biological processes that involve cellular heterogeneity, including stem/progenitor cell maintenance/differentiation [43–45], T cell recruitment/expansion [46, 47], cancer clonal evolution [48], tumor-initiating potential [49], metastatic potential [50], and response to targeted therapies [51, 52]. In this study, we utilized clonal tracing and mathematical modeling to dissect cancer clonal constitution in response to ICB treatment. We performed DNA barcoding of mouse cancer cells to label each cell and its progeny with a unique barcode and examined the clonal constitution of cancer cells under ICB treatment in different hosts. To interrogate the cancer cell-intrinsic contribution to ICB resistance, we compared the frequency of each clone in different treatment groups. To interrogate the cancer cell-extrinsic contribution to ICB resistance, we compared the clonal constitution of each tumor harvested from different mice with the same ICB treatment. Based on these comparisons, we further evaluated the cancer cell-intrinsic and cancer cell-extrinsic signatures of immune evasion in response to ICB treatment.

## Results

### Performance of biomarkers for ICB response is inconsistent across clinical trials

**Fig. 1:**
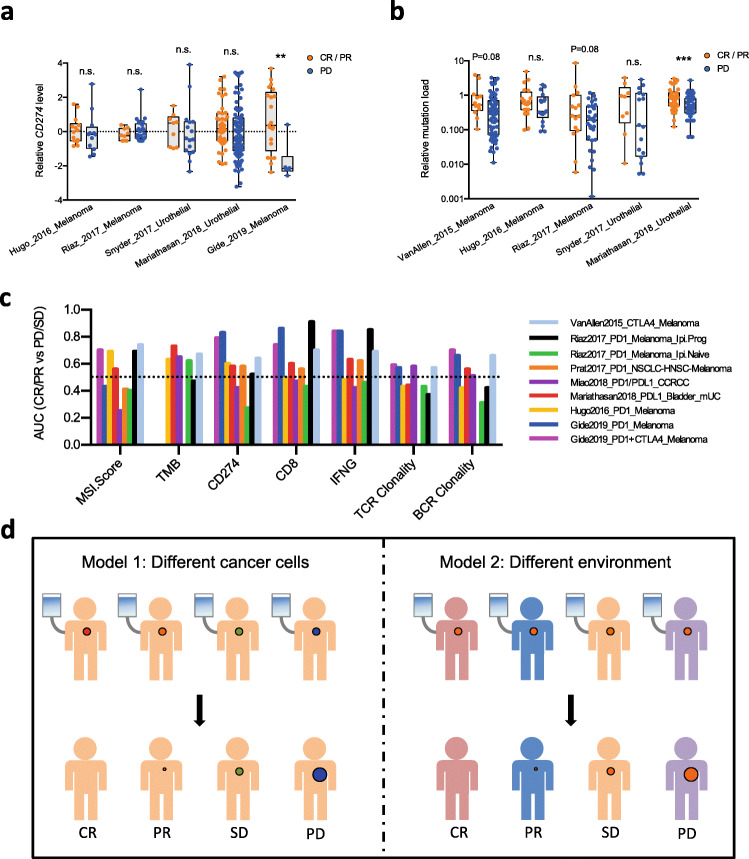
Performance of biomarkers is incoherent across different ICB clinical cohorts. **a** *CD274* level significantly correlates with ICB response in Gide et al., but not the other studies. **b** Tumor mutation burden significantly correlates with ICB response in the Mariathasan et al. study, but not in the Hugo et al. or Snyder et al. studies (boxplot shows the minimum, first quartile, median, third quartile, and maximum values of each group; n.s., not significant; **P < 0.01, ***P < 0.001; Student’s *t* test with Benjamini-Hochberg adjustment of *P* values for multiple comparison). **c** Systematic evaluation of multiple biomarkers of ICB response in different clinical cohorts reveals inconsistent performance. **d** Two non-mutually exclusive models can explain the inconsistent performance of biomarkers in different clinical cohorts. Model 1 assumes that different mutation profiles and epigenetic status of cancer cells from different tumors (colored dots) determine the heterogeneous response (size of dots) after ICB treatment (syringe). Model 2 assumes that host-specific factors determine response

Multiple clinical studies have been performed to address the efficacy of ICB in multiple cancer types and investigate the biomarkers of response for optimal stratification of patients for treatment. Pre-treatment PD-L1 level was proposed as a biomarker for better response to nivolumab, an anti-PD-1 antibody [1]. Higher tumor mutation burden and DNA mismatch repair deficiency were also identified to correlate with better ICB response [2, 3]. We performed a closer assessment of such biomarkers in recent clinical cohorts, which revealed inconsistent performance in segregating ICB responders and non-responders (Fig. 1a, b). For example, higher *CD274* mRNA level significantly correlates with better ICB response in the Gide et al. study [53], but not in multiple other studies that used anti-PD-1 or anti-PD-L1 [20, 35, 54, 55] (Fig. 1a). Consistent with this, a literature search also revealed variable prediction power of PD-L1 protein level on anti-PD-1 response in different studies [3, 56]. Similarly, higher tumor mutation load significantly correlates with better response in the Mariathasan et al. study [35], but not in multiple other studies [20, 55] (Fig. 1b). We systematically evaluated other frequently reported biomarkers in existing clinical cohorts, including microsatellite instability (MSI) score, *CD8a*/*CD8b*, *IFNG*, and intra-tumoral T cell or B cell clonality. All of these biomarkers showed highly variable performance in predicting the likelihood of response to ICB (Fig. 1c).

There are two complementary models that can explain such inconsistent performance (Fig. 1d). In the first model, the different genetic mutation profiles and epigenetic status of cancer cells between different tumors determine the different responses to immunotherapy. According to this model, each clinical cohort samples a small number of patients out of the vast number of potential combinations of mutation profiles and epigenetic status, and the biomarkers correlated with response in a certain trial occur by chance, with the observations not repeated in other trials. This model is substantiated by studies identifying different cancer cell-intrinsic mechanisms of immune resistance [12, 57] and indicates that larger sample sizes are needed to achieve reliable biomarker performance. The second model assumes that even for the same cancer cells grown in different patients, the response can still be diverse due to tumor microenvironment or other host factors. If the second model is correct, a combination of biomarkers measuring orthogonal aspects of the tumor would be required for a reliable prediction of response. Although both models may hold true, with each partially explaining the response to ICB in any given patient, the second model is more difficult to test in human clinical studies because every naturally occurring human tumor is genetically and contextually distinct. We therefore established a novel mouse system to address this problem.

### Establishing an in vivo system to assess the heterogeneity in ICB response

To study the response patterns to ICB treatment, we first used the mouse CT26 colorectal cancer cell line model, which shows moderate response to anti-PD1 or anti-CTLA4 treatment with remarkable heterogeneity [58]. We used the ClonTracer barcoding system [51] to uniquely label clones in CT26 cell populations for in vivo study. We implemented several stringent criteria during transduction to ensure unique labeling of each transduced cell with a distinct barcode. First, we used a much lower number of CT26 cells for transduction compared to the total complexity of the ClonTracer barcode library, which minimizes the chance of labeling different cells with the same barcode. Second, we performed transduction at very low multiplicity of infection (MOI = 0.01), which lowers the chance of infecting one cell with multiple virions. After transduction, CT26 cells carrying barcodes were selected and expanded in vitro, and high-throughput sequencing was performed to evaluate barcode representation in the population of cells. We found that barcoded cells from the same transduced batch showed good consistency in barcode identity and frequency, whereas those from different batches showed minimal overlap (Additional file 1: Fig. S1), indicating good reproducibility and uniqueness of our barcode approach. To guarantee sufficient coverage of each barcoded cancer clone for in vivo experiments, we selected the batch of transduced cells with ~ 2000 distinct barcodes for further study.

**Fig. 2:**
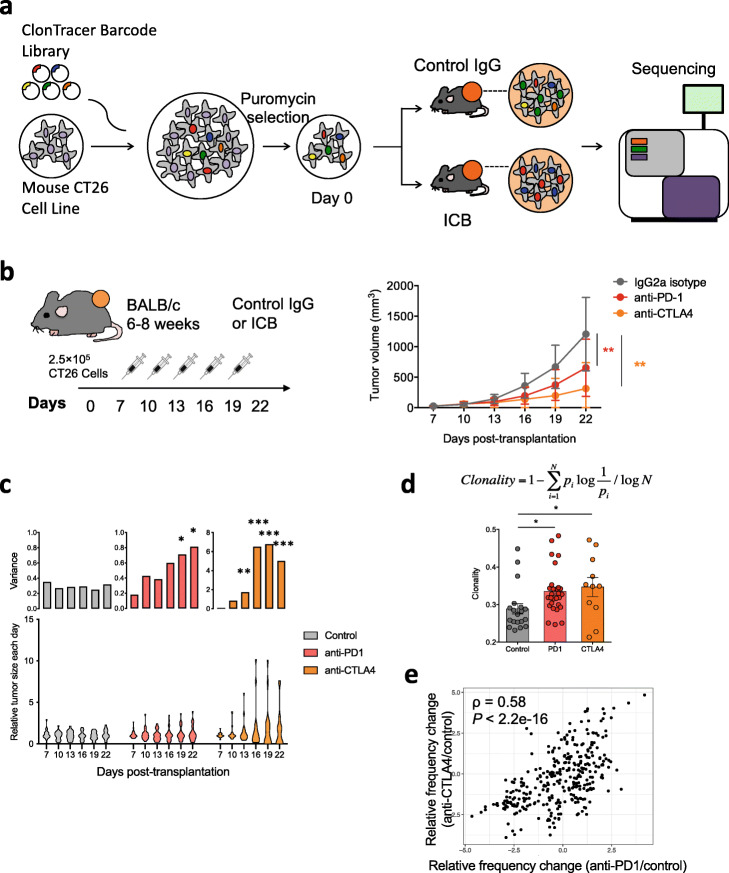
Clonal barcoding reveals heterogeneity in response to ICB. **a** Experimental design for clonal barcoding. We first transduced the parental CT26 line with the high-diversity ClonTracer barcode library at a low MOI (0.01). We selected and expanded the transduced cells (containing ~ 2000 distinct barcodes), and transplanted into syngeneic recipients, with 250,000 barcoded cells each flank site, two sites per recipient. We then treated the recipient mice with control IgG (*N* = 10), anti-PD-1 (*N* = 15), or anti-CTLA-4 (N = 10). After treatment, we harvested tumor for barcode quantification. **b** Anti-PD-1 or anti-CTLA-4 treatment (syringes) significantly reduces tumor growth compared to control IgG treatment (mean ± SD; *P < 0.05, ***P < 0.001; two-way ANOVA with Bonferroni post-test multiple comparison). **c** Distribution of relative tumor size (lower panel, normalized to the median value in each group) and its intra-group variance (upper panel) for the control IgG, anti-PD-1, and anti-CTLA-4 groups along the treatment course. ICB treatment led to significantly higher intra-group variance (*P < 0.05, ***P < 0.001; *F* test of equality of variance). **d** Clonality of barcode distribution in the anti-PD1 or anti-CTLA4 group is significantly higher than that in the control IgG treatment group (mean ± SD; *P < 0.05; one-way ANOVA with Bonferroni post-test multiple comparison). **e** The enrichment/depletion of barcodes between anti-PD1 and anti-CTLA4 is positively correlated

We transplanted 250,000 in vitro expanded barcoded cells per site into syngeneic recipient Balb/c mice subcutaneously (2 sites per mouse) and allowed the tumors to establish in vivo for 7 days. We then treated the recipient mice with control IgG, anti-PD-1, or anti-CTLA-4, and observed significantly reduced tumor growth upon ICB treatment (Fig. 2a, b). ICB treatment significantly increased the intra-group variance in tumor size (Fig. 2c), indicating individual heterogeneity in ICB response. In addition, we examined this finding in multiple other syngeneic transplantation models (MC38, 4T1, and EMT6) and found consistently that ICB treatment led to higher variance in tumor size in each model (Additional file 1: Fig. S2a-c).

To evaluate the barcode constitution in each tumor, we PCR-amplified and sequenced the DNA barcodes from each remaining tumor and from cells cultured in vitro. Compared to the in vitro samples, over 80% of clones were consistently absent in the in vivo samples, suggesting strong engraftment selection (Additional file 1: Fig. S2d-e). The in vivo barcodes showed significantly higher clonality in the anti-PD-1- or anti-CTLA-4-treated tumors compared to control IgG-treated tumors, indicating clonal selection by ICB treatment (Fig. 2d). The clones enriched in the anti-PD-1 treatment also tended to be enriched in anti-CTLA-4 treatment, suggesting shared cancer cell-intrinsic ICB-resistance mechanisms for the two regimens (Fig. 2e).

Our observations on CT26 syngeneic tumors treated with anti-PD-1 or anti-CTLA-4 suggest heterogeneous response at both the bulk tumor level and at the cancer clone level. At the bulk tumor level, different tumors initiated by the same clones of cancer cells exhibit diverse growth dynamics in response to ICB. At the cancer clone level, different clones within the same tumor show heterogeneous sensitivity to ICB treatment. Based on these results, we further examined whether the bulk tumor-level heterogeneity is associated with clonal level heterogeneity.

### ICB responders had stronger clonal selection and higher immune infiltration than non-responders

**Fig. 3:**
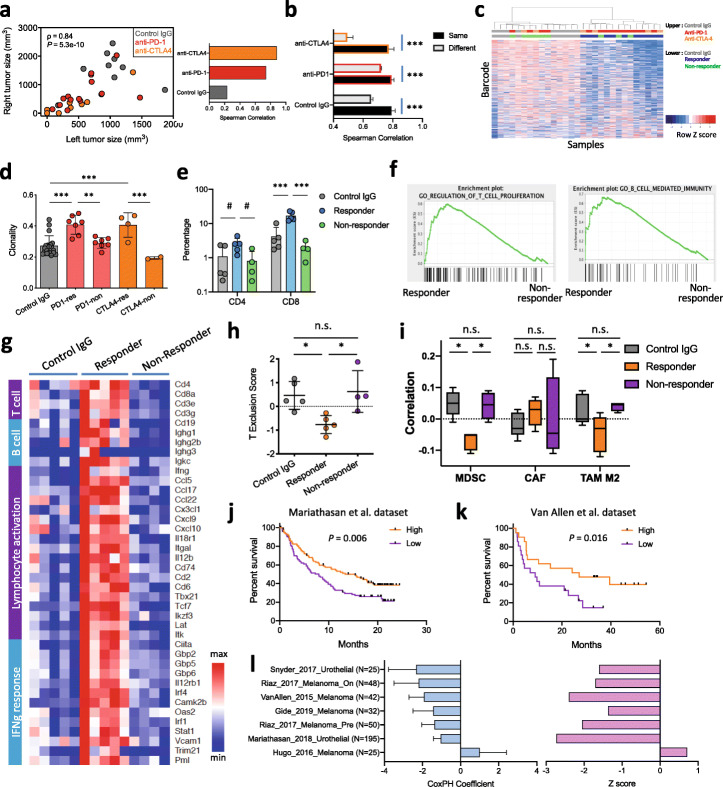
ICB responders and non-responders show different clonal response patterns. **a** The size of tumors from the same mouse is positively correlated. **b** Correlation of barcode distribution of tumors from the same mouse is significantly higher than that from different mice. **c** Hierarchical clustering of in vivo tumor samples based on barcode distribution. Generally, responders cluster separately from non-responders and control-treated tumors. Each row represents a specific barcode, and each column represents a tumor sample. To assist visualization, rows were ordered by the difference between the responders and the control IgG group. **d** Clonality of barcode distribution is significantly higher in ICB responders (mean ± SD; **P < 0.01, ***P < 0.001; one-way ANOVA with Benjamini-Hochberg post-test multiple comparison). “res” are responders and “non” are non-responders. **e** Summary of percentage of intra-tumoral CD4+ and CD8+ cells in control IgG, ICB responders, or non-responders (mean ± SD; ^#^*P* < 0.1, ***P < 0.001; one-way ANOVA with Benjamini-Hochberg post-test multiple comparison). **f** GSEA analysis of bulk tumor RNA-seq reveals higher expression of genes involved in T cell proliferation or B cell-mediated immunity in responders compared to non-responders. The entire list of enriched gene sets can be found in Additional file 3: Table S2. **g** Heatmap of relative expression of the top differentially expressed genes. Within the gene sets that were enriched, we picked representative genes to reflect the differential enrichment of gene sets. Responders had higher expression of genes in adaptive immunity. **h** TIDE reveals higher T cell exclusion scores in non-responders than responders (mean ± SD; *P < 0.05; n.s., not significant; one-way ANOVA with Benjamini-Hochberg post-test multiple comparison). **i** Correlation of bulk tumor expression in each group with MDSC, cancer-associated fibroblast (CAF), or M2 macrophage. Responders showed lower correlation with MDSC and M2 macrophage than control IgG or non-responders (mean ± SD; *P < 0.05; n.s., not significant; one-way ANOVA with Benjamini-Hochberg post-test multiple comparison). **j**, **k** Differential gene expression signature in ICB responder from our study correlates with better survival in the Mariathasan et al. (**j**) or Van Allen et al. (**k**) studies. *P* values were derived using Cox-PH regression treating signature score as a continuous variable. **l** Cox-PH regression coefficient value (mean ± SEM) and *z* score of the signature score in its correlation with survival hazard in multiple ICB treatment clinical trials

The finding that different tumors composed of the same cancer clones responded differently to ICB treatment indicates a cancer-extrinsic mechanism of response from the host. Indeed, comparing the tumors that developed on the left and right flanks on each recipient mouse, we found their tumor size to be significantly positively correlated, especially in the anti-PD-1- and anti-CTLA-4-treated groups (Fig. 3a). In addition, barcode distribution in the two tumors from the same mouse showed significantly higher correlation than those in tumors from different mice (Fig. 3b). These results indicate that systemic factors at the host level may influence tumor growth in both control IgG- and ICB-treated groups.

To investigate why different ICB responses arose between tumors derived from the same batch of cancer cells, we identified ICB responders and non-responders by tumor size post-treatment (Additional file 1: Fig. S3a-b) and compared their clonal distributions and tumor microenvironments. Hierarchical clustering and a Kolmogorov-Smirnov test based on barcode distribution showed that the ICB responders had distinct clonal constitution of cancer cells compared to control IgG-treated mice (Fig. 3c, Additional file 1: Fig. S3c). In addition, the ICB responders had higher clonality of barcode distribution, in other words, higher unevenness in barcode distribution, than the non-responders (Fig. 3d, Additional file 1: Fig. S3d). Further examination of the tumor microenvironment by IHC revealed that the responders had higher infiltration of T cells in the tumors (Fig. 3e, Additional file 1: Fig. S3e). This prompted us to further compare the responders and the non-responders by bulk tumor RNA-seq. Principal component analysis (PCA) revealed that the ICB responders had distinct gene expression from the non-responders (Additional file 1: Fig. S3f). Consistent with observed higher immune infiltration from IHC, the responder tumors had higher expression of genes involved in antigen binding, adaptive immune response, and lymphocyte-mediated immunity than the non-responders (Fig. 3f, g, Additional file 1: Fig. S3g), and lower expression of genes involved in protein translation and oxidative phosphorylation (Additional file 1: Fig. S3h). The responders also showed higher expression of pro-inflammatory cytokines/chemokines, such as *Ifng*, *Cxcl9*, *Cxcl10*, and *Ccl5* (Fig. 3g). Furthermore, we utilized another mouse model (MC38 colorectal cancer) to investigate the difference between ICB responders and non-responders. Again, we observed strong association between primary response to ICB and cancer cell-extrinsic factors (Additional file 1: Fig. S3i).

Intra-tumoral TCR clonality was reported to be associated with ICB response in recent studies [59]. We therefore applied the TRUST [60] algorithm on the tumor RNA-seq data to infer the tumor-infiltrating immune repertoires. Different from regular TCR/BCR-seq approaches that sequence the immune repertoires directly from the genomic DNA, TRUST extrapolates immune repertoires from RNA-seq data. Responders had higher levels of recombined immune repertoires (Additional file 1: Fig. S4a-b), potentially due to their higher level of adaptive immune infiltration. Consistent with previous studies, we detected slightly higher TCR clonality and significantly higher BCR clonality in the responders (Additional file 1: Fig. S4c), supporting the role of clonal expanded T and B cells in tumor elimination. Furthermore, we applied our previously published computational method TIDE [41] on the tumor RNA-seq data to estimate the level of immunosuppressive cell types that can cause T cell exclusion (Fig. 3h, i). Consistent with the IHC and immune marker gene expression, T cell exclusion score is higher in non-responders than responders (Fig. 3h), showing positive correlation with gene expression signatures from myeloid-derived suppressor cells (MDSC) and M2 macrophages (Fig. 3i). We assessed the clinical relevance of the response-associated gene expression signature in mouse by assessing the association between signature score and patient survival in published ICB treatment cohorts with Cox-PH regression. We found that the mouse responder signature significantly correlated with a lower survival hazard (reflected by a negative Cox-PH regression coefficient value and *z* score) in the majority of ICB clinical studies (Fig. 3j–l). Together, these results show that cancer cells originating from the same ancestors can form tumors of ICB responders or non-responders.

### ICB treatment enriched ICB-resistant cancer clones in responders

**Fig. 4:**
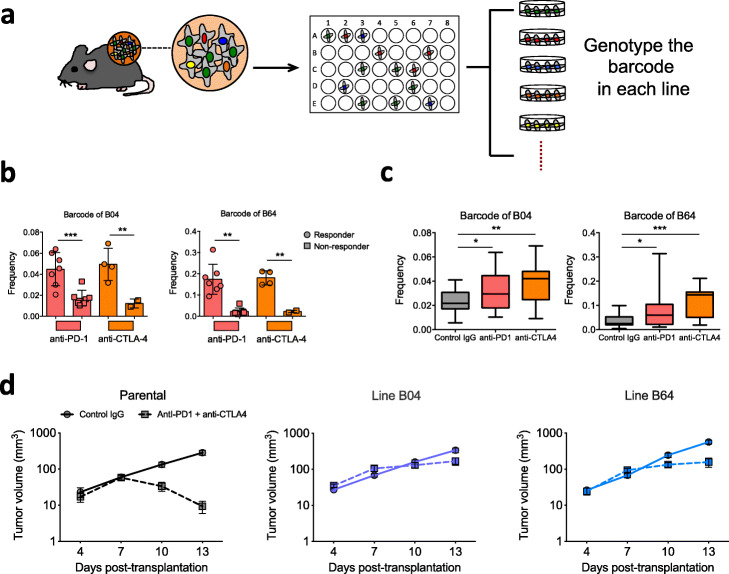
ICB treatment enriched ICB-resistant cancer clones in responders. **a** Design for isolation of ICB-resistant clone. Tumors (large blue dot) were dissociated and cancer cells (small blue dots) were plated into 96-well plates at limiting dilution. Single cell-occupied wells were selected by microscopy and then expanded to establish stable lines. We genotyped each established line by Sanger sequencing of the barcode and inferred its ICB sensitivity based on enrichment/depletion by anti-PD1 and/or anti-CTLA4. **b** Frequency of the clones representing line B04 or line B64 is higher in responders than non-responders of ICB (mean ± SD; **P < 0.01, ***P < 0.001; one-way ANOVA with Benjamini-Hochberg post-test multiple comparison). **c** Frequency of the barcodes representing line B04 or line B64 is higher in the anti-PD1- or anti-CTLA4-treated group than the control group (boxplot shows the minimum, first quartile, median, third quartile, and maximum values of each group; *P < 0.05, **P < 0.01, ***P < 0.001; one-way ANOVA with Benjamini-Hochberg post-test multiple comparison). **d** Response to combined ICB treatment confirmed the ICB resistance of lines B04 and B64. We transplanted 250,000 cells (parental, line B04, or line B64) into the syngeneic recipients and treated the tumor with control IgG or combinatorial anti-PD1 + anti-CTLA4 on days 4, 7, and 10 post-transplantation. Tumors derived from the parental line decreased in size after ICB treatment, whereas those derived from line B04 or line B64 persisted

The assays above addressed the heterogeneity of response to ICB treatment at the bulk tumor level. We then focused on the response at the cancer clone level by isolating single cell-derived clones to investigate their phenotype (Fig. 4a).

We established ~ 150 single cell-derived lines from engrafted tumors by limiting dilution. Upon genotyping of the barcode in each line, we identified 5 lines (numbered A01, A06, B04, B64, and C32) representing 2 distinct barcoded clones that were significantly enriched in the ICB responders compared to the non-responders (Additional file 2: Table S1, Fig. 4b, Additional file 1: Fig. S5). These clones were also enriched in both anti-PD-1- and anti-CTLA-4-treated tumors compared to the IgG-treated control (Fig. 4c), suggesting they are more resistant to ICB treatment than the average of other clones. To verify this, we conducted another round of growth experiments on the B04 and B64 lines in vivo (each represented by different barcodes) and compared these to the parental line. The “parental line” used here and later experiments refers to the in vivo-derived bulk CT26 cells to control for engraftment-mediated selection and facilitate data comparison. Lines B04 and B64 manifested increased resistance to combined anti-PD-1/anti-CTLA-4 (Fig. 4d), demonstrating that the cancer clones enriched in the ICB responders were more resistant to ICB treatment. These results suggest that the ICB responders had a higher composition of ICB-resistant cancer clones following ICB treatment. Together with the microenvironment characterization in Fig. 3, our data support a model that the responders had higher cancer cell-extrinsic antitumor immunity, and such selective pressure consequently drove clonal selection and enrichment of cancer cell-intrinsic ICB-resistant clones. In contrast, the non-responders lacked effective T cell infiltration and showed a lower level of clonal selection.

### ICB-resistant clones show distinct transcription signatures associated with T cell dysfunction

We tried to understand the mechanisms of ICB resistance in clones B04 and B64 from different aspects. First, loss of antigen presentation or sensitivity to IFNγ induction has been reported to confer resistance to immunotherapy [12, 16, 61]. We therefore compared the MHC-I and PD-L1 levels of B04, B64, and the parental line, but observed similar baseline levels and similar induction by IFNγ (Additional file 1: Fig. S6a-b). This indicates that the resistance mechanisms of B04 and B64 are not due to lack of MHC-I expression or insensitivity to IFNγ.

**Fig. 5:**
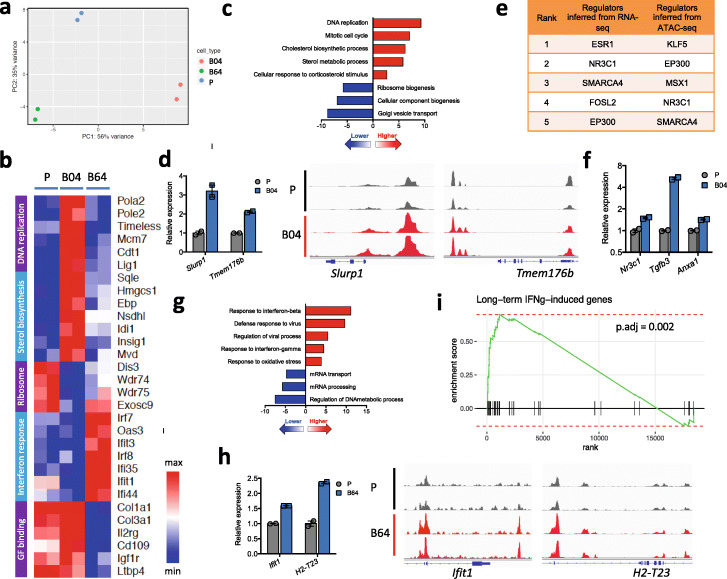
ICB-resistant clones show different transcriptional signatures associated with T cell dysfunction. **a** PCA of RNA-seq data from parental line CT26 and ICB-resistant lines B04 and B64 suggests distinct expression profiles between the three lines. **b** Heatmap of relative expression of the top differentially expressed genes between the resistant lines and the parental line. **c** Cistrome GO enrichment of pathways up- or downregulated in line B04 compared to the parental line, integrating RNA-seq and ATAC-seq data. **d** Immune response-related genes *Slurp1* and *Tmem176b* have higher RNA expression (barplot on the left) and genomic DNA accessibility (alignment plot on the right) in line B04 compared to the parental line. **e** Top transcription regulators in line B04 compared to the parental line, inferred from RNA-seq (by LISA) or ATAC-seq (by CistromeDB). **f** GR and its targets are expressed higher in line B04 compared to the parental line. Each individual value and mean ± SD are plotted for each group. **g** Cistrome GO enrichment of pathways up- or downregulated in line B64 compared to the parental line, integrating RNA-seq and ATAC-seq data. **h** Immune response-related genes *Ifit1* and *H2-T23* have higher RNA expression (barplot on the left) and genomic DNA accessibility (alignment plot on the right) in line B64 compared to the parental line. **i** GSEA shows upregulation of genes induced by long-term IFNγ induction (gene set derived from Benci et al. [15]). Each vertical black line represents a gene upregulated by long-term IFNγ induction in the Benci et al. study

Next, we investigated whether B04 and B64 lines are associated with T cell dysfunction. To this end, we borrowed insights from our previous TIDE study [41], which utilized a Cox proportional hazard (Cox-PH) model to identify gene expression signatures associated with T cell dysfunction in TCGA and PRECOG datasets. This signature reflects the gene expression in both the dysfunctional T cells and in other cells in the tumor which are associated with T cell dysfunction. We conducted RNA-seq analyses on B04, B64, and the parental line and observed a significant positive correlation between differentially expressed genes in the resistant lines and the TIDE T cell dysfunction signature (Additional file 1: Fig. S7a). Genes with a strong positive TIDE dysfunction *z* score, whose higher expression indicates cytotoxic T cell dysfunction, were upregulated in the ICB-resistant lines (Additional file 1: Fig. S7b), whereas those with negative TIDE *z* score were unchanged or downregulated (Additional file 1: Fig. S7c). While this observation suggested that cancer cells with B04 and B64 signature might be associated with T cell dysfunction in clinical tumors, we wondered whether they might represent distinct mechanisms. Indeed, PCA of the RNA-seq profiles revealed that these two resistant lines have distinct gene expression changes compared to the parental line (Fig. 5a). Closer examination revealed that line B04 had higher expression of genes in DNA replication and sterol biosynthesis and lower expression of genes in ribosome biogenesis, whereas line B64 had higher expression of genes in interferon response and lower expression of genes in growth factor binding (Fig. 5b). We performed VarScan analysis of RNA-seq data from parental CT26 line, line B04, and line B64 to identify the genetic differences. We found a mutation in *Arhgap30* (Rho GTPase Activating Protein, L458S) that was present in B04 but absent in parental line and B64. We did not find any B64-specific mutations. *Arhgap30* was expressed in B04 but not expressed at detectable levels in parental line or B64, which likely explains why this SNP was detected only in B04. Although this gene is reported to be correlated with LDL cholesterol levels, we do not know what underlies the differential expression of this gene. We therefore speculate that the transcriptional differences in B04 and B64 might be driven by epigenetic factors.

To further characterize the differentially expressed genes in the resistant lines, we performed ATAC-seq to assess their differences in chromatin accessibility. Similar to RNA-seq, PCA on the ATAC-seq data also suggests distinct epigenetic changes between line B04 and line B64 as compared to the parental line (Additional file 1: Fig. S7d). Using regulatory potential derived from our previously developed BETA algorithm [62] to assign the differential ATAC-seq peaks to genes, we found good correlation between differential ATAC-seq and differential RNA-seq (Additional file 1: Fig. S7e-f). We then used Cistrome GO [63] to find the most differentially regulated genes by combining the differential ATAC-seq and differential RNA-seq data. The upregulated genes in line B04 were enriched for cell cycle and sterol metabolic pathways from GSEA [63, 64] analysis (Fig. 5c) and included many genes known to be related to immune response. For example, *Slurp1* and *Tmem176b* were upregulated in line B04 (Fig. 5d). SLURP1 is a secreted protein with immunomodulatory effects [65], and TMEM176B is a cation channel protein only recently discovered to suppress antitumor immunity via the inflammasome pathway [66]. To infer the putative transcription factors regulating the observed differential ATAC-seq and differential RNA-seq, we used two computational methods to exploit published ChIP-seq and chromatin accessibility profiles in Cistrome Data Browser (CistromeDB) [67]. For differential ATAC-seq, we used the toolkit function in CistromeDB [68] to identify transcription regulators whose published binding sites have the highest overlap with our differential ATAC-seq peaks. For differential RNA-seq, we used LISA [69] to first construct a chromatin model based on the differential genes and then identify the transcription regulators whose binding has the biggest effect on these genes based on the chromatin model. The two approaches retrieved consistent predictions, with nuclear receptor NR3C1, SWI/SNF complex component SMARCA4, and histone acetyltransferase EP300 as top candidates (Fig. 5e, Additional file 4: Table S3, Additional file 5: Table S4). In support of the NR3C1 prediction, the expression of *Nr3c1* (encoding glucocorticoid receptor, GR) as well as its downstream targets was significantly upregulated in B04 (Fig. 5f). GR is known to mediate the immunosuppressive effects of glucocorticoids, and its agonists have been used clinically to suppress inflammation and relieve autoimmune side effects [70–72]. GR ChIP-seq from CistromeDB also revealed that GR can bind to the promoter and/or enhancer regions of *Slurp1* and *Tmem176b* (Additional file 1: Fig. S7g) [73]. Activation of GR in cancer cells was reported to induce resistance to chemotherapy or targeted therapies [74–76], and our results suggest that GR might also induce resistance to immunotherapy.

Different from our observations in line B04, the most upregulated genes from combined differential RNA-seq/ATAC-seq data in line B64 were enriched for genes involved in response to type I and type II interferons (Fig. 5g), including genes *Ifit1* and *H2-T23* (Fig. 5h). IFIT1 is an interferon-stimulated gene with reported antiviral effects [77], and H2-T23 is a class Ib MHC that interacts with the NKG2A inhibitory receptor on NK cells and T cells to suppress their activation [78, 79]. In addition, key transcription factors downstream of interferon, such as *Irf7*, *Irf8*, and *Stat2*, were upregulated in B64 and can bind to the promoter/enhancer region of *Ifit1* and *H2-T23* (Additional file 1: Fig. S7h-j). Recently, multiple studies reported that type I and II interferon signaling can compromise cancer cell response to T cell-mediated killing [15, 80], via mechanisms such as upregulation of T cell inhibitory receptor ligands (e.g., PD-L1 and MHC-II) and other cancer-associated inhibitory interferon-stimulated genes (e.g., *IFIT1* and *MX1*). Indeed, the genes induced by long-term IFNγ treatment in their study which were reported to be associated with ICB resistance were also enriched in the upregulated genes in line B64, including *Ifit1* (Fig. 5h, i).

In summary, these results suggest that different cancer clones, even those derived from the same parental line, can have distinct transcriptomic and epigenetic characteristics, and heterogeneous sensitivity to ICB treatment. These clones are subject to different selection pressures in different hosts, which likely underlie the diverse response patterns observed.

### Mathematical modeling of the clonal dynamics predicts that ICB-resistant clones are enriched in responders

**Fig. 6:**
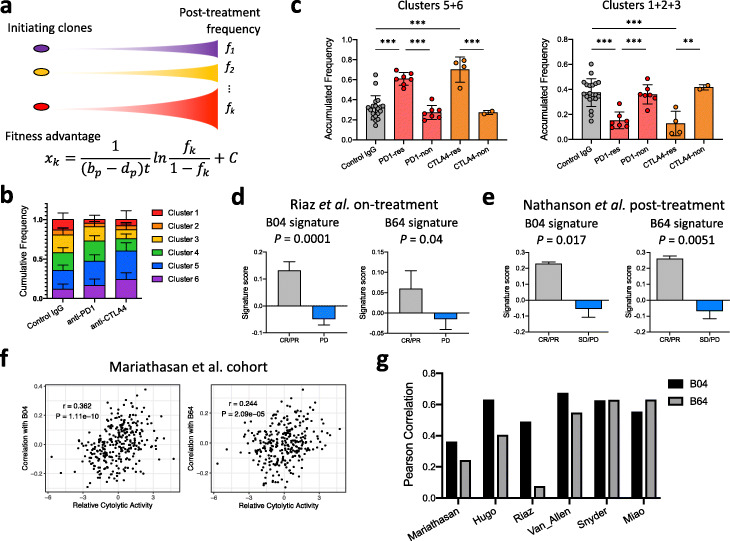
Mathematical modeling reveals little contribution of ICB-resistant clones to the ICB resistance by the bulk tumor. **a** Scheme of the mathematical modeling of tumor clonal constitution. The fitness advantage of a clone (*x*_*k*_) can be expressed in a formula containing the proliferation and death rate of the bulk population (*b*_*p*_ and *d*_*p*_, respectively), time of growth (*t*), and frequency of this clone at tumor harvest (*f*_*k*_). **b** The cumulative frequencies of clones belonging to each cluster learned from the mathematical model in groups treated by control IgG, anti-PD-1, or anti-CTLA4 (mean ± SD). **c** The cumulative frequency of clones belonging to cluster 5 or 6 in **b** is significantly higher in ICB responders. The cumulative frequency of clones belonging to cluster 1, 2, or 3 in **b** is significantly higher in non-responders (mean ± SD; **P < 0.01, ***P < 0.001; one-way ANOVA with Benjamini-Hochberg post-test multiple comparison). “res” are responders and “non” are non-responders. **d**, **e** Cancer cell-intrinsic resistance signatures derived from line B04 or B64 significantly correlate with better ICB response within **d** on-treatment samples in the Riaz et al. study [54] and **e** post-treatment samples in the Nathanson et al. study [82] (mean ± SD; two-sided *t* test). **f**, **g** Cancer cell-intrinsic resistance signatures derived from line B04 or B64 correlate with intra-tumoral cytolytic activity from multiple clinical studies

Our results above demonstrated that there are heterogeneous responses to anti-PD-1 and anti-CTLA-4 not only at the bulk tumor level but also at the clonal level. To quantitatively assess the contribution of clonal level ICB resistance to bulk tumor growth, we performed mathematical modeling of the barcoded clones in the tumors. We first established the theoretical framework of clonal selection in vivo by deducing the growth advantage of a clone compared to the bulk population [81]. This model assumed that the fitness advantage of each clone is reflected by its log-transformed frequency (Fig. 6a). We applied a Bayesian statistical inference method [83] to model clone selection under all treatment conditions and identified 6 clusters of clones with different growth patterns and sensitivity to ICB treatment (Fig. 6b, Additional file 1: Fig. S8a-c). Among them, there are clones more sensitive to both anti-PD-1 and anti-CTLA-4 (clusters #1, #2, and #3), and those more resistant to both anti-PD-1 and anti-CTLA-4 (clusters #5 and #6). Consistent with experimental validation of resistance, lines B04 and B64 were assigned to the resistant clusters #5 and #6, respectively.

To examine the contribution of ICB-resistant clones to the ICB response in the bulk tumors, we compared the cumulative frequency of clones in each cluster among different tumors. Consistent with the observation for lines B04 and B64, resistant clones (those belonging to clusters #5 and #6) showed higher frequency in ICB responders than non-responders (Fig. 6c). Conversely, sensitive clones (those belonging to clusters #1, #2, and #3) showed higher frequency in non-responders than responders (Fig. 6c). When expanding the analysis to all tumors, we observed a negative correlation between ICB-resistant clone abundance and tumor size after ICB treatment (Additional file 1: Fig. S8d), and conversely a positive correlation between ICB-sensitive clone abundance and tumor size (Additional file 1: Fig. S8e). These results revealed that the non-responders may actually contain a higher percentage of ICB-sensitive cancer cells after ICB treatment. It also implies that tumors with cancer cell-intrinsic resistance signatures in the on-treatment samples may correlate with better tumor response to ICB, suggesting that the tumors harbor sufficient immunity to eliminate the ICB-sensitive cancer cells. Interestingly, tumors treated by control IgG showed the same trend as those treated by ICB (Additional file 1: Fig. S8d-e), indicating that pre-existing intra-tumoral immune pressure might impose clonal selection of cancer cells even in the absence of ICB treatment.

To validate the clinical relevance of these observations from the mouse tumor model, we examined the bulk tumor RNA-seq data from multiple clinical studies [11, 20, 35, 54, 82, 84]. In the two studies where both pre-treatment and on-treatment tumor RNA-seq data are available for comparison [54, 82], the B04 and B64 gene expression signatures are significantly associated with better ICB response in the on/post-treatment tumors (Fig. 6d, e), but not the pre-treatment ones (Additional file 1: Fig. S8f-g). This is consistent with our observation in the mouse that the resistant clones were enriched after response to ICB treatment. Furthermore, in other studies where only pre-treatment RNA-seq data is available, B04 and B64 signatures show positive correlation with microenvironmental cytolytic activity based on transcription of *GZMA* and *PRF1* [85] (Fig. 6f, g). This observation supports our computational modeling that the presence of cancer cell-intrinsic immune resistance signature in tumors prior to immunotherapy treatment is an indication of pre-existing immune pressure and cytolytic activities.

Taken together, our approach combining a unique mouse system and extensive clinical data mining from multiple cohorts demonstrates that tumors originating from the same group of cancer cells can show diverse response patterns to ICB. Therefore, in addition to mutation profiling at diagnosis, characterizing cancer clonal constitution and the tumor microenvironment during treatment may provide complementary insights to improve patient outcomes.

## Discussion

Heterogeneous response to ICB among different patients remains a major challenge of cancer immunotherapy. Numerous clinical studies have been performed to assess treatment efficacy and response biomarkers [1–3, 10, 11, 20, 53, 54]. However, it is difficult to identify consistent resistance patterns, likely due to the remarkable inter- and intra-tumor heterogeneity. In this study, we identified heterogeneous responses to ICB treatment in multiple syngeneic transplantable tumor models and further identified diverse response patterns of the same cancer clones in tumors with different responses to ICB.

Comparing tumor growth and barcode distribution across different mouse tumors within the same treatment group, we demonstrated that response can differ dramatically between tumors with the same initial clonal constitution. Good response to ICB is strongly associated with elevated microenvironmental immune infiltration and T cell activation. Such microenvironment-dominated signature is associated with good prognosis in all but one clinical cohort tested. Comparing barcode frequencies between different treatment groups followed by clonal validation, we found that ICB-resistant cancer clones exist in the bulk cancer cell population prior to ICB treatment. We isolated two ICB-resistant cancer clones for further study and discovered that they have different transcriptional and epigenetic profiles and harbor distinct ICB-resistance mechanisms. Line B04 has elevated expression of genes downstream of nuclear receptors, such as GR. Glucocorticoid signaling was reported to mediate diverse effects in both cancer cells and immune cells. GR agonists, such as dexamethasone, can inhibit the activation and proliferation of lymphocytes, thus suppressing inflammation [70, 71, 86]. On the other hand, elevated GR signaling in cancer cells can mediate resistance to targeted therapy and promote cancer metastasis [74–76, 87]. Further investigation of GR is needed to uncover its role in immunotherapy response. Line B64 has elevated basal level of interferon response signature. This was surprising because interferon signaling is critical for T cell-driven inflammation and response to ICB treatment [12, 16], so increased interferon response signature was not expected to be associated with ICB resistance. However, recent studies also revealed that prolonged interferon exposure can regulate a multigenic resistance program to ICB [15], and indeed, we found genes involved in such resistance program to be upregulated in B64. In addition, elevated interferon signaling has also been reported to mediate resistance to chemotherapy, radiotherapy, or targeted therapies [88–90].

Built upon barcode distributions, our mathematical modeling revealed that ICB responders, but not non-responders, may show enrichment of cell-intrinsic ICB-resistance signatures during ICB therapy. Our simulation result comports with analyses of clinical data that ICB responders, rather than non-responders, show an enrichment of cancer cell-intrinsic resistance signature post-treatment. Consequently, the presence of ICB-resistant cancer clones is more likely to contribute to the late-developing resistance in the initial ICB responders, rather than the primary resistance in the initial non-responders. This finding marks a critical difference between immunotherapy and targeted therapies and emphasizes the critical role of cancer cell-extrinsic factors in ICB response. Systematic examination of the abundance of ICB-resistant clones and characterization of tumor microenvironment in ICB responders or non-responders corroborates the mathematical simulation and confirms the critical role of tumor microenvironment in ICB response. Our result indicates that genetic biomarkers alone may not be able to completely distinguish ICB responders from non-responders. Instead, an expression signature that reflects both the cancer cells and the immune microenvironment might provide important insights to ICB response prediction.

Despite the intriguing observations from our study, it remains elusive how different tumor microenvironments arise between different recipients of the same cancer cell line. Previous studies revealed multiple cancer cell-intrinsic mechanisms that can modulate the tumor microenvironment [14, 21, 57, 91–94]. However, in our study, we transplanted the same cancer cell line, so the difference in microenvironment between ICB responders and non-responders is unlikely attributable to cancer cell-intrinsic factors. Other studies suggested that the gut microbiota in each individual may play a critical role in their immune system development and ICB response [37, 95, 96]. In our study, all mice were obtained from the same company and maintained in the same facility, and we did not observe significant differences between cages, so the difference in ICB response we observed might not be attributable to the gut microbiota. One possible explanation is the general immune health of each individual at the time of tumor establishment and ICB treatment, a comprehensive state influenced by diet, exercise, immune repertoire, mental health, and other factors which merit further study. Another limitation of our study is that we isolated the single cell-derived cancer clones after, rather than before, ICB treatment. This is because all cancer clones had very low initial frequencies and ICB treatment provided a means to enrich ICB-resistant clones for isolation. Consequently, the transcriptome and epigenetic changes in lines B04 and B64 might be either pre-existing prior to ICB treatment or induced by ICB treatment. In either scenario, our data indicate that there are several pathways by which cancer cells may become ICB-resistant. Furthermore, as is the limitation with most cancer models in mice, the speed of disease development in transplantation models usually precludes the feasibility of assessing the role of mutation accumulation in the process of tumor development and ICB response. We expect that genomic engineering tools can be integrated with these models to simulate mutation accumulation and clonal evolution along tumor development and treatment response.

Whereas both cancer cell-intrinsic factors and cancer cell-extrinsic factors are known to influence the response to immunotherapy, our study found that even for the same labeled cancer cells grown in isogenic individuals, the immune response can be diverse. This finding has two important implications for clinical treatment and translational research. First, our results indicate that there is an inherent limitation in using pre-treatment genetic information to guide immunotherapy treatment decisions because tumors with a defined genetic background may show heterogeneous responses. Therefore, longitudinal data on the tumor microenvironment, such as by RNA-seq of biopsies, or monitoring of patient general immune health, such as by examining peripheral blood, will likely help optimize treatment strategies. Second, our study reveals a paradoxical observation, which may affect interpretations of tumor RNA-seq data from studies associating ICB response with expression signatures. When studying biomarkers for ICB response or resistance, it is common to use RNA-seq from tumors that respond to ICB as the “responder” signature. Our study suggests that immune-resistant cancer cells are enriched in such samples. Therefore, the “responder” signature derived this way may in fact be enriched for a cancer cell-intrinsic resistance signature, the opposite of the intended result. This phenomenon may thereby contribute to the variable performance of ICB response biomarkers in different clinical cohorts.

## Conclusions

In this study, we sought to address the heterogeneity of response to ICB. From analysis of published ICB clinical trials, we found that each biomarker shows variable prediction power in different studies. To further assess the source of such heterogeneity, we performed clonal tracing and mathematical modeling of cancer clonal dynamics in response to ICB. We found that the same population of cancer clones can show diverse response patterns in different tumors. ICB responders showed higher immune infiltration and enrichment of ICB-resistant clones post-treatment compared to non-responders. The cancer cell-intrinsic ICB-resistance signatures in on-treatment/post-treatment samples are correlated with better ICB response. These results demonstrate that tumors originating from the same cancer cells can show heterogeneous responses to ICB depending on the tumor microenvironment; therefore, longitudinal data on the tumor microenvironment or patient immune health may complement mutation profiling to optimize treatment. Furthermore, because ICB responders may be enriched for cancer cell-intrinsic resistant signatures, our study suggests that deconvolution of tumor RNA-seq data into cancer cell-intrinsic/extrinsic signatures may be required for proper interpretation of response-signature association studies.

## Methods

### Mice

All mice were housed in standard cages in Dana-Farber Cancer Institute Animal Resources Facility (ARF). All animal procedures were carried out under the ARF Institutional Animal Care and Use Committee (IACUC) protocol and were in accordance with the IACUC standards for the welfare of animals. Wild-type Balb/c and C57BL/6 recipient mice were purchased from Charles River laboratory.

### Barcoding of cancer cells

CT26 mouse colorectal cancer cell line was cultured in RPMI-1640 medium supplemented with 10% FBS and 1% penicillin-streptomycin. We transduced the CT26 cells with the ClonTracer barcode retroviral library using 8 μg/ml polybrene. After 24-h incubation, infected cells were selected with puromycin. To ensure that the majority of cells were transduced with a single virion, we performed the transduction at low multiplicity of infection (MOI < 0.05). To avoid marking multiple clones with the same barcode, we restricted the number of cells infected during the transduction (< 10,000) prior to the follow-up expansion (Supplementary Fig. 1a). Expanded cultures from the same batch of infection were highly consistent in barcode distribution (Supplementary Fig. 1b), enabling systematic assessment of enrichment or depletion of clones in response to drug treatment.

### Barcode amplification and sequencing

At the end of ICB treatment in vivo, we harvested residual tumors from each recipient mouse and extracted bulk genomic DNA with the AllPrep DNA/RNA Mini Kit (Qiagen) for barcode amplification. We used PCR to amplify the barcode sequence for NGS with protocols as previously described [51]. We sequenced the PCR-amplified barcode library on the Illumina HiSeq-2500 sequencer, with paired-end 150 TruSeq kit. Deconvolution of sequencing results and quantification of barcode composition were performed by clonTracer_v1.2 software [51].

### Assess the heterogeneity of ICB response between different tumors and different cancer clones

We transplanted 2.5 × 10^5^ barcoded CT26 cells subcutaneously into the left and right flank of 6–8-week-old female Balb/c mice (from Charles River Laboratory). When the tumors became palpable (day 7 post-transplantation), we began treatment with control IgG (clone 2A3), anti-PD1 (clone 1A12), or anti-CTLA4 (clone 9D9), by intraperitoneal injection (200 μg per mouse in 200 μl HBSS buffer) every 3 days for a total of 5 times. Three days after the last treatment, we harvested tumors for immunohistochemistry and DNA/RNA extraction.

To assess the heterogeneity of ICB response between different tumors, we monitored tumor size along the course of treatment and identified “responder” or “non-responder” tumors in the anti-PD1 or anti-CTLA4 cohorts based on their size at the end of treatment compared to the tumors in the control IgG cohort. Specifically, tumors larger than 10^*M* − 0.1^ were classified as “non-responder” and tumors smaller than 10^*M* − 0.5^ were classified as “responder” (*M* denotes the logarithmic median tumor size in the control IgG cohort). We also applied an alternative approach to classify responder and non-responder by the magnitude of change between post- and pre-treatment tumor size, which led to similar results. We then performed immunohistochemistry and bulk tumor RNA-seq to characterize the difference between responders and non-responders. Notably, there were 8 tumors in the anti-CTLA4 cohort with complete response, which prevented us from profiling their tumor microenvironment.

To evaluate the intra-tumoral heterogeneity of cancer clones in response to ICB treatment, we used the genomic DNA harvested from each tumor at the end of treatment course for barcode amplification and sequencing. We compared global barcode composition in all samples by hierarchical clustering. For each barcode, we compared its log-transformed frequency in control IgG, anti-PD1, or anti-CTLA4 cohort by ANOVA.

### Assess the heterogeneity of ICB response in other syngeneic models

A total of 250,000 MC38, 4T1, or EMT6 cells were transplanted subcutaneously (for MC38) or into the mammary fat pad (for 4T1 and EMT6) into syngeneic recipients. When the tumors grew to 100–200 mm^3^ in size, we started control IgG or anti-PD1 treatment 100 μg per mouse every 3 days for a total of 4 doses. We monitored tumor size along the treatment course and compared the intra-group variance between the control IgG and anti-PD1 groups in each model by the *F* test of the equality of variances.

### Isolation of ICB-resistant single cell-derived clones

We first transplanted 2.5 × 10^5^ barcoded CT26 cells into syngeneic Balb/c recipient mice for tumor establishment in vivo. On day 16 post-transplantation, after the tumor was established in vivo, we harvested the bulk tumor for isolation of single cell-derived clones and then genotyped each clone for their barcode identity. Specifically, tumors were cut into small pieces, digested in collagenase/hyaluronidase for 20 min at 37 °C, washed with RPMI media, and filtered through a 70-μm cell strainer. Cells were resuspended in RPMI medium supplemented with 10% FBS and 1% penicillin/streptomycin for culture. The next day, we started treatment of the cell culture with 10 μg/ml puromycin for 2 days to select barcoded CT26 cells.

To acquire single cell-derived clones, we plated the barcoded CT26 cells from the previous step into 96-well plates at limiting dilution and selected wells occupied by a single cell under the microscope for further culture. We then extracted genomic DNA from each single cell-derived clone and sequenced its barcode for identification. Each single cell-derived clone was evaluated for ICB sensitivity/resistance based on the depletion/enrichment of its corresponding barcode by anti-PD1 or anti-CTLA4 treatment.

### Validation of ICB resistance in isolated lines

As above, we cultured bulk barcoded CT26 cells isolated from established tumors as control “parental” line and expanded the resistant lines #B04 and #B64, which harbored the barcodes enriched by both anti-PD1 and anti-CTLA4 treatment. To verify the ICB resistance of line B04 and line B64, we transplanted 2.5 × 10^5^ parental, line B04, or line B64 cells into 6–8-week-old Balb/c recipient mice and performed combination ICB or single agent treatment for validation. For single agent treatment, we used 200 μg control IgG (clone 2A3), anti-PD1 (clone 1A12), or anti-CTLA4 (clone 9D9) per mouse each time. For anti-PD1/anti-CTLA4 combination treatment, we used 200 μg control IgG per mouse in the control treatment group and 100 μg anti-PD1 + 100 μg anti-CTLA4 in the ICB treatment group per mouse each time.

All mouse studies were approved by IACUC at Dana-Farber Cancer Institute and performed in accordance with the IACUC standards for the welfare of animals.

### Flow cytometry

For assessment of MHC-I and PD-L1 levels, CT26 cells (in vivo-selected parental control or isolated ICB-resistant lines) were treated by vehicle control or 10 ng/ml IFNγ for 48 h. Cells were then dissociated and resuspended in PBS-2%FBS and incubated with DAPI, anti-PD-L1 (clone MIH5, BD Biosciences), and anti-H2-K^d^ (clone SF1-1.1, BioLegend) for 1 h on ice. Cells were then washed, resuspended in PBS-2%FBS, and analyzed on BD LSR-Fortessa instrument. FACS data was then analyzed by FlowJo software.

For assessment of intra-tumoral immune infiltration in the MC38 model, 9-week-old C57BL/6 (Charles River) mice were implanted with 1 × 10^5^ MC38 cells in each flank. On days 14, 16, and 18 post-tumor implantation, animals received intraperitoneal injection of anti-PD-1 monoclonal antibody (clone 29F.1A12, 50 μg/animal). Animals were euthanized on day 32 post-tumor implantation, and the resulting tumors were dissected, digested (Miltenyi mouse tumor dissociation kit, 130-096-730), depleted of red blood cells by ACK solution lysis, and stained with fluorescent conjugated antibodies for flow cytometry analysis for tumor-infiltrating immune cell populations using an established antibody panel and flow cytometry protocol [97]. We used the summed frequency of CD4+ and CD8+ T cells in all viable singlet cells to quantify T cell infiltration in each sample.

### Immunohistochemistry

Immunohistochemistry of FFPE samples of mouse tumors was performed at the Brigham and Women’s Hospital (BWH) Specialized Histopathology Core Lab.

### RNA-seq

For comparison of transcription profile between single cell-derived clones and the parental line, we extracted total RNA from in vivo-selected parental line or isolated ICB-resistant lines (#B04 and #B64) cultured in complete RPMI-1640 culture medium in duplicates. RNA extraction was performed using the miRNeasy Plus Mini Kit (Qiagen, #17004) following the manufacturer’s protocol. Total RNA was submitted to Novogene Inc. for sequencing. Standard mRNA library preparation kit was used for library preparation. Paired-end 150 bp sequencing was done on Illumina HiSeq 2500. RNA-seq fastq reads were aligned to the mm10 genome using STAR v2.5 [98] with default settings and quantified using RSEM [99] with default settings. The downstream differential gene expression analysis was performed using DESeq2 [100], with default settings, and differentially regulated gene sets were derived by GSEA [64] with default settings. To identify genetic mutations in the barcoded cell lines, we performed VarScan [101] analysis of parental CT26 line, line B04, and line B64 RNA-seq with default settings.

For comparison of transcription profile between tumor samples, we extracted total RNA from bulk tumor samples using the AllPrep DNA/RNA Mini Kit (Qiagen, #80204). Total RNA was submitted to Novogene Inc. for sequencing as above.

### ATAC-seq

For comparison of chromatin accessibility between single cell-derived clones and the parental line, ATAC-seq was performed using in vivo-selected parental line or isolated ICB-resistant lines (#B04 and #B64) in duplicates (200,000 cells) by the Center for Functional Cancer Epigenetics at DFCI as previously described. For data analysis, we used Burrows-Wheeler Aligner (BWA) to map sequencing reads to the reference genome and MACS2 for peak calling. DESeq2 was applied to identify the differentially accessible regions between ICB-resistant lines and the parental control line.

### Infer transcriptional regulators from RNA-seq

We inferred the transcriptional regulators underlying the transcriptional difference between the parental line and ICB-resistant lines by the LISA algorithm (http://lisa.cistrome.org/). Briefly, given a differentially expressed gene set, LISA uses DNase-seq and H3K27ac ChIP-seq data in public databases to model the chromatin landscape of the cell type and lineage context. Based on the chromatin landscape, LISA uses two methods to infer the relevant transcription regulators by evaluating all the ChIP-seq-determined transcription regulator binding sites.

With direct ChIP method, for each transcription regulator ChIP-seq dataset, LISA examines the regulatory potential for the target gene set compared to background gene set. The transcription regulators with largest difference in regulatory potential between target and background gene sets are reported as the driving transcription regulators.

With in silico deletion method, LISA first selects a small number of DNase-seq or H3K27ac ChIP-seq samples from the CistromeDB based on chromatin regulatory potential to discriminate the target gene set from a random background gene set. It then evaluates each putative transcription regulator cistrome by first setting the DNase-seq/H3K27ac ChIP-seq chromatin signal to zero on the cistrome peaks and then testing whether it influences regulatory potential for target and background gene set. The transcription regulators that influence target gene set more than background gene set are reported as the driving transcription regulators.

The above two methods generated very similar results for our differentially expressed gene sets. We report the results for in silico deletion method in this manuscript.

### Infer transcriptional regulators from ATAC-seq

We inferred transcriptional regulators associated with the differential ATAC-seq peaks by testing the significance of overlap with publicly available ChIP-seq peaks for each candidate transcription regulator (http://dbtoolkit.cistrome.org/).

### Integrative analysis of RNA-seq and ATAC-seq

We integrated the results of RNA-seq and ATAC-seq to identify significantly regulated biological processes by Cistrome GO (http://go.cistrome.org/). Briefly, Cistrome GO first ranks genes by magnitude of change in transcription (using RNA-seq results) or change in regulatory potential (using ATAC-seq) and then integrates the two rankings for each gene into one ranking score. Finally, Cistrome GO performs GSEA analysis on integrated gene ranking for enriched GO terms.

### Gene set enrichment analysis

GSEA was performed by software from Broad Institute, with gene sets from MSigDB as reference.

### Extrapolation of immune repertoire

We extracted the CDR3 sequence and relative abundance of T/B cell receptors TRA/TRB and IGH/IGL/IGK by TRUST [60, 102].

### TIDE T cell dysfunction/exclusion signature scores

We calculated T cell dysfunction and exclusion scores by TIDE as previously described [41]. For T cell dysfunction scores, we used the Cox-PH model to test how the expression of a candidate gene interacts with the level of cytotoxic T cell infiltration in affecting death hazard. For T cell exclusion scores, we calculated the Spearman correlation of each sample’s transcriptome with the reference transcriptome of MDSC, M2 macrophage, or cancer-associated fibroblast.

### Mathematical modeling of the cancer clonal dynamics in vivo

We established a theoretical framework to simulate the selection of ICB-resistant clone, adapting a published model on subclonal selection in cancer [81]. We denote the proliferation rate and death rate of the bulk cancer cell population as *b*_*p*_ and *d*_*p*_, respectively. For a specific cancer clone *k*, we denote the proliferation and death rate as *b*_*k*_ and *d*_*k*_, respectively. The fitness advantage of clone *k* (*x*_*k*_) can be defined by the ratio of net growth rates between this clone and the background host population:

1 $$ 1+{x}_k=\frac{b_k-{d}_k}{b_p-{d}_p} $$

When *x* > 0, the corresponding cancer clone has growth advantage over the average of the bulk population, under the condition tested.

We denote the frequency of the ICB-resistant clone as *f*_*k*_ and the time of growth in vivo as *t*. The frequency of clone *k* at time of tumor harvest can be deduced as:

2 $$ {f}_{k\left(\mathrm{end}\right)}=\frac{f_{k\left(\mathrm{start}\right)}\cdotp {e}^{\left({b}_k-{d}_k\right)t}}{f_{k\left(\mathrm{start}\right)}\cdotp {e}^{\left({b}_k-{d}_k\right)t}+\left(1-{f}_{k\left(\mathrm{start}\right)}\right)\cdotp {e}^{\left({b}_p-{d}_p\right)t}} $$

Because several hundred clones successfully engrafted in each mouse, we can assume that *f*_*k*(start)_ << 1. Combining Eqs. (1) and (2), we can deduce the value for relative growth advantage as:

3 $$ {x}_k=\frac{1}{\left({b}_p-{d}_p\right)t}\left(\mathit{\ln}\frac{f_{k\left(\mathrm{end}\right)}}{1-{f}_{k\left(\mathrm{end}\right)}}+\mathit{\ln}\frac{1}{f_{k\left(\mathrm{start}\right)}}\right) $$

For a specific cancer clone *k*, its frequency in the tumor at the start of treatment should be the same across different treatment groups. According to Eq. (3), the fitness advantage of this clone under each treatment condition should be proportional to $\mathit{\ln}\frac{f_{k\left(\mathrm{end}\right)}}{1-{f}_{k\left(\mathrm{end}\right)}}$.

### Detection of different growth patterns

We fitted the frequencies of barcoded cancer cells into a finite mixture of linear regression models to capture the detailed tumor heterogeneity. Our barcoding experiment ensured that each barcode represented a single clone and clones with the same genomic profile could be labeled by multiple barcodes. Assume that there are *K* unique clonotypes in the tumor:

4 $$ h\left(y|x,\psi \right)={\sum}_{k=1}^K{\pi}_kf\left(y|x,{\theta}_k\right),s.t.{\pi}_k\ge 0,{\sum}_{k=1}^K{\pi}_k=1 $$

where the cell growth rate *y* depends on cell intrinsic and extrinsic factors *x* with the density function *h*(), and *ψ* includes the parameter estimations on the prior probability *π*_*k*_ of component *k* and the component-specific parameter *θ*_*k*_. Since we repeatedly measured the barcode changes with *N*_*m*_ observations from barcode *m*, the log-likelihood of the mixture model becomes:

5 $$ \log L={\sum}_{m=1}^M{\sum}_{n=1}^{N_m}\log h\left({y}_{mn}|{x}_{mn},\psi \right),\kern0.5em {\sum}_{m=1}^M{N}_m=N $$

We used the FlexMix package in R to optimize the log-likelihood and compute the posterior probability of barcode *m* belonging to clonotype *k*. We define that the log of barcode frequency follows the Gaussian distribution with linear factors from the treatment condition and the log of tumor size.

### Evaluate the correlation of cancer cell-intrinsic or cancer cell-extrinsic signatures to clinical ICB response

Similar to the TIDE T cell dysfunction/exclusion estimation, we calculated the line B04/B64 signature scores of bulk tumors as the Pearson correlation between bulk tumor gene expression and line B04/B64 gene expression. We first defined B04 (or B64) gene signature as the log_2_-fold-change of genes in each line compared to the parental line. For each tumor within one clinical cohort (e.g., the Riaz pre-treatment cohort), we normalized log_2_-TPM of gene *i* in sample *j* as the deviation from the mean log_2_-TPM of gene *i* in all samples in the cohort:

$$ {E}_{i,j}^{\prime }={E}_{i,j}-\overline{E_i} $$

To derive the B04 (or B64) signature score in a tumor sample, we calculated the Pearson correlation between the reference signature (the log_2_-fold-change from the first step) and the normalized gene expression in the tumor sample (${E}_{i,j}^{\prime }$). If a sample has high expression of genes that are also highly expressed in line B04 (or B64), and low expression of genes that are also lowly expressed in line B04 (or B64), then this sample should have a high B04 (or B64) signature score. We used the 200 most up/downregulated genes in B04 and B64 for signature score calculation in order to focus on the genes that define the difference between these lines and the parental line and eliminate the noise that can be introduced by the genes with low expression variance. For pre-treatment or on-treatment samples in each clinical cohort, we compared the Pearson correlation values between CR/PR patients and PD patients by Student’s *t* test. For pre-treatment samples, we also compared the Pearson correlation coefficients and the cytolytic activity score of each patient and found a significantly positive correlation in the majority of studies. We performed robustness test (e.g., using the top 200, 400, 800, or all significantly differentially expressed genes between line B04/B64 and the parental line) and found the conclusion to be robust under different parameters.

Similarly, for cancer cell-extrinsic factors, we first calculated the differential gene expression between ICB responders and non-responders and included the top 200 most regulated genes to get the cancer cell-extrinsic signature. We computed the Pearson correlation between the cancer cell-extrinsic signature and the transcriptome of each patient in each clinical cohort. For each cohort, we calculated the association between patient survival and signature score (treated as a continuous variable) by Cox-PH regression.

## Supplementary information

**Additional file 1.** Supplementary Figures.

**Additional file 2: Table S1.** Barcode identity of the isolated single-cell-derived clones.

**Additional file 3: Table S2.** Enriched pathways in responders vs non-responders by GSEA.

**Additional file 4: Table S3.** Cistrome Toolkit inference of transcription regulators with highest overlap with differential ATAC-seq peaks.

**Additional file 5: Table S4.** LISA inference of transcription regulators that may best account for the differentially expressed genes.

**Additional file 6.** Review history.

## Data Availability

All sequencing data were uploaded to Gene Expression Omnibus with accession number GSE139476 [103]. All analysis codes were uploaded to the GitHub repository: https://github.com/Shengqing-Stan-Gu/2020_Clone_Tracing_paper [104] and Zenodo: 10.5281/zenodo.4016933 [105]. The source code is licensed under the MIT license. The following public datasets were used: Mariathasan [35], Van Allen [11], Riaz [54], Snyder [55], Miao [84], Gide [53], Hugo [20], Nathanson [82], and Benci [15].
