# Supplementary material for: Clonal tracing reveals diverse patterns of response to immune checkpoint blockade
Source: Genome Biol. 2020 Oct 15;21:263. doi: 10.1186/s13059-020-02166-1 (PMC7559192; doi:10.1186/s13059-020-02166-1)
Supplement: Supplementary file 1 — Additional file 1. Supplementary Figures. [file 13059_2020_2166_MOESM1_ESM.docx]

**Supplementary Figures**


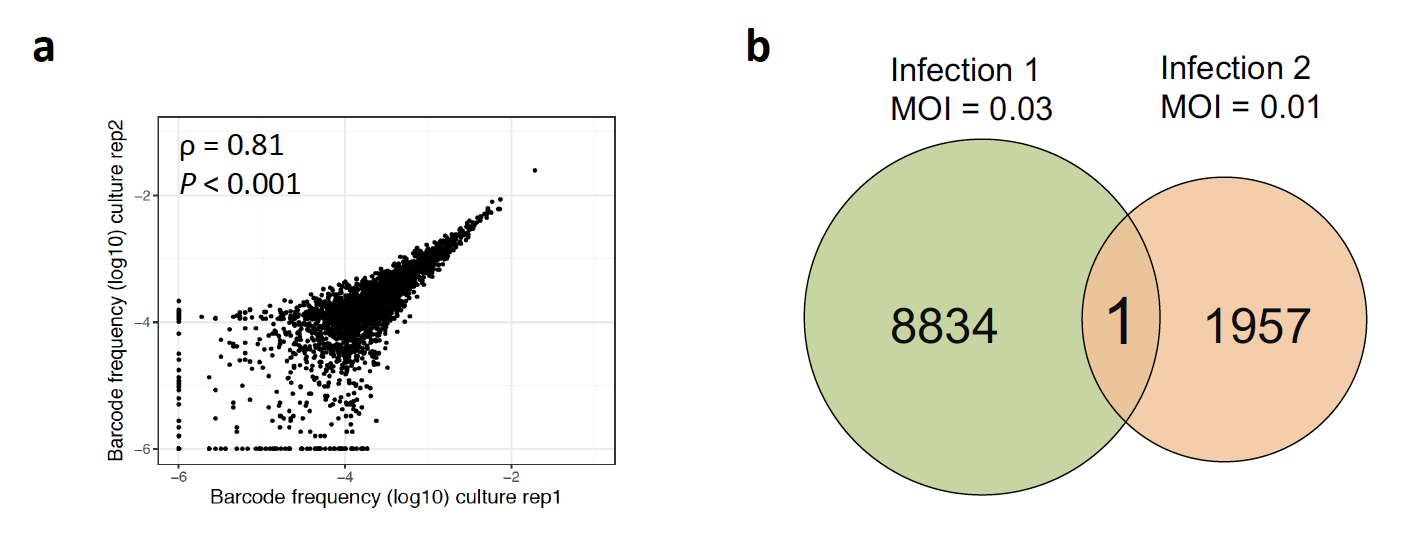


**Fig. S1. Quality control of ClonTracer barcoding system.**

(a) Barcode frequencies of two replicates of cell culture derived from the same batch of infection show strong positive correlation. (b) Venn diagram of transduced barcode numbers from two independent infections. Distinct barcodes were transduced with minimal overlap, indicating that the barcode library diversity far exceeds the number of infected cells in each infection.


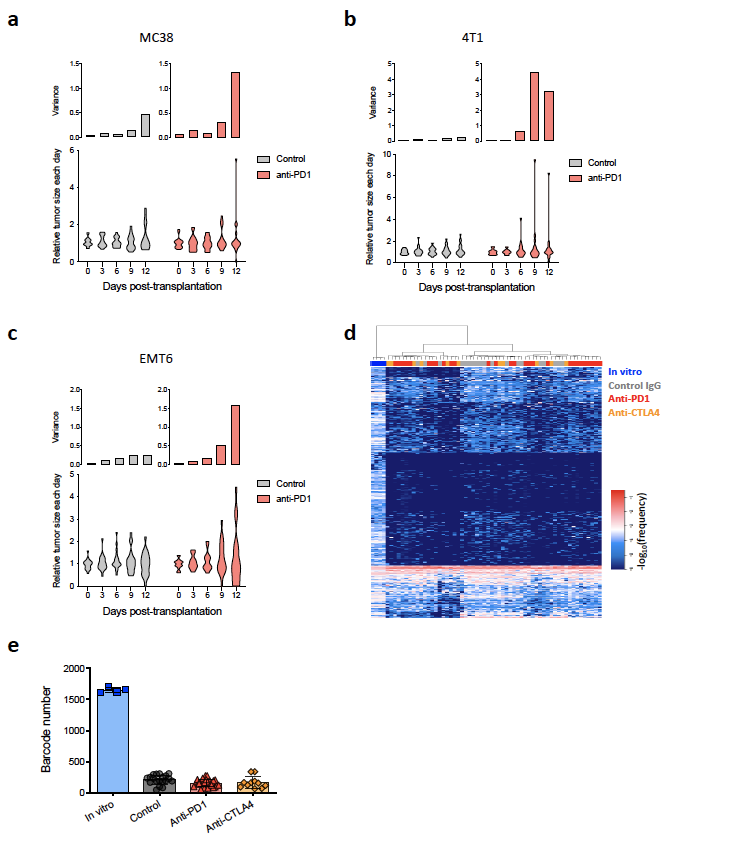


**Fig. S2. Heterogeneity of growth at bulk tumor level and at clonal level.**

(a-c) Distribution of relative tumor size (normalized to the median value in each group) and its intra-group variance for the control IgG and anti-PD-1 groups for (a) MC38, (b) 4T1, and (c) EMT6 along the treatment course. (d) Hierarchical clustering of in vitro and in vivo samples based on barcode constitution. In vivo samples clustered together, distinct from in vitro samples. However, in vivo samples with different treatment were not well separated. (e) Number of barcodes identified in each sample from: *in vitro* culture (N = 4), tumor treated by control IgG (N = 19), tumor treated by anti-PD1 (N = 28), or tumor treated by anti-CTLA4 (N = 11).


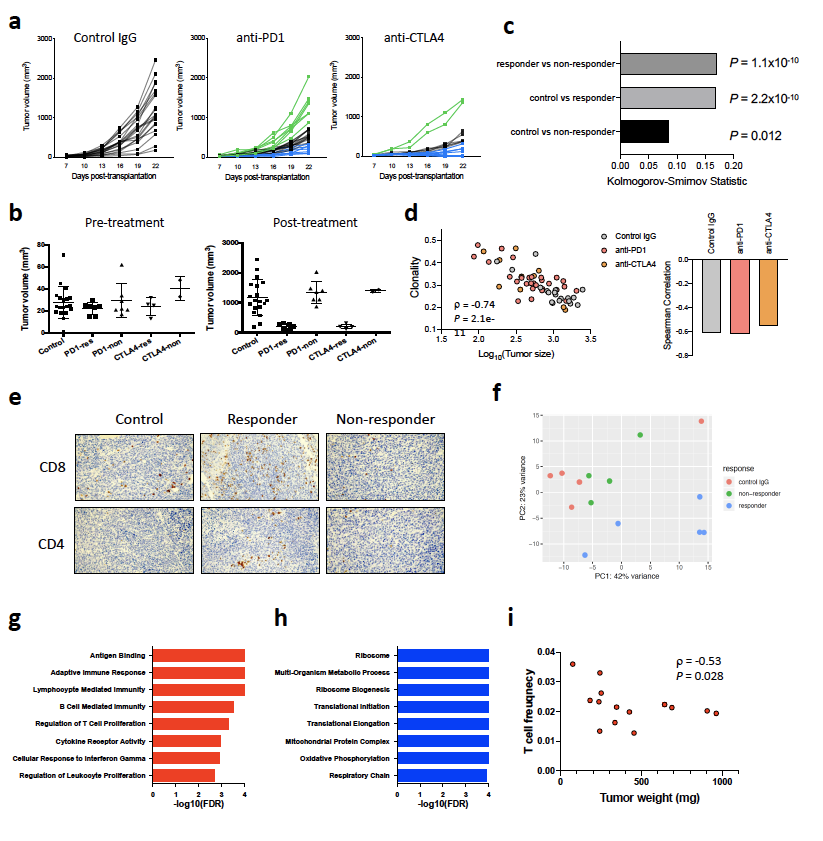


**Fig. S3. ICB responders and non-responders show distinct microenvironmental gene expression.**

(a) Individual tumor growth curves under the treatment of control IgG, anti-PD1, or anti-CTLA4. Tumors colored in blue were classified as responders while tumors colored in green were classified as non-responders. (b) Pre-treatment and post-treatment tumor size of control IgG treated tumors, ICB responders, or non-responders. (c) Kolmogorov-Smirnov test of barcode distribution between control IgG, ICB responder, and non-responder groups. (d) Clonality of barcode distribution negatively correlates with the post-treatment tumor size in all treatment groups. (e) Representative immunohistochemistry images of CD8 or CD4 cells in control IgG treated tumors, ICB responders, or non-responders. (f) PCA of RNA-seq data suggests distinct expression profiles between the ICB-responders and non-responders. (g-h) GSEA enrichment of GO terms in genes with (g) increased or (h) reduced expression in ICB responders. (i) In the MC38 model where recipients were treated by anti-PD-1, the intratumoral T cell infiltration showed a negative correlation with tumor mass.


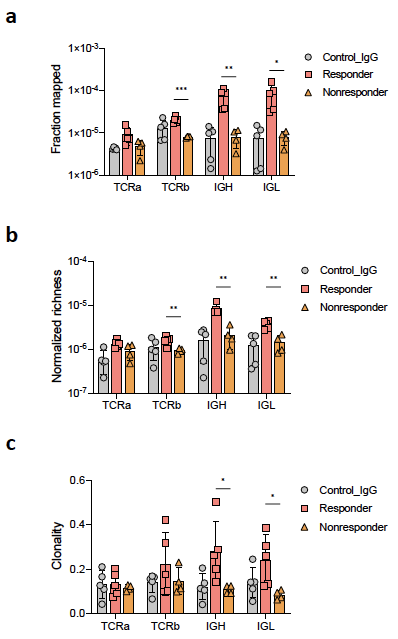


**Fig. S4. Characteristics of tumor-infiltrating TCR/BCR repertoire.**

(a) Percentage of bulk tumor RNA-seq reads that were mapped to *TRA*, *TRB, IGH, or IGL* genes. There are significantly higher levels of BCR/TCR reads in ICB responders. (mean ± s.d.; * *P* < 0.05, ** *P* < 0.01, *** *P* < 0.001; t-test between responder and non-responder) (b) Immune repertoire from ICB responders has higher richness normalized to sequencing depth than that from non-responders. (mean ± s.d.; ** *P* < 0.01; t-test between responder and non-responder) (c) Comparison of immune repertoire clonality between ICB responders and non-responders. To account for the level of immune infiltration as a confounding factor, we calculated the clonality of immune clonotypes that constituted the top 90% in abundance in each sample. ICB responders had higher intratumoral clonality than non-responders. (mean ± s.d.; * *P* < 0.05; t-test between responder and non-responder)


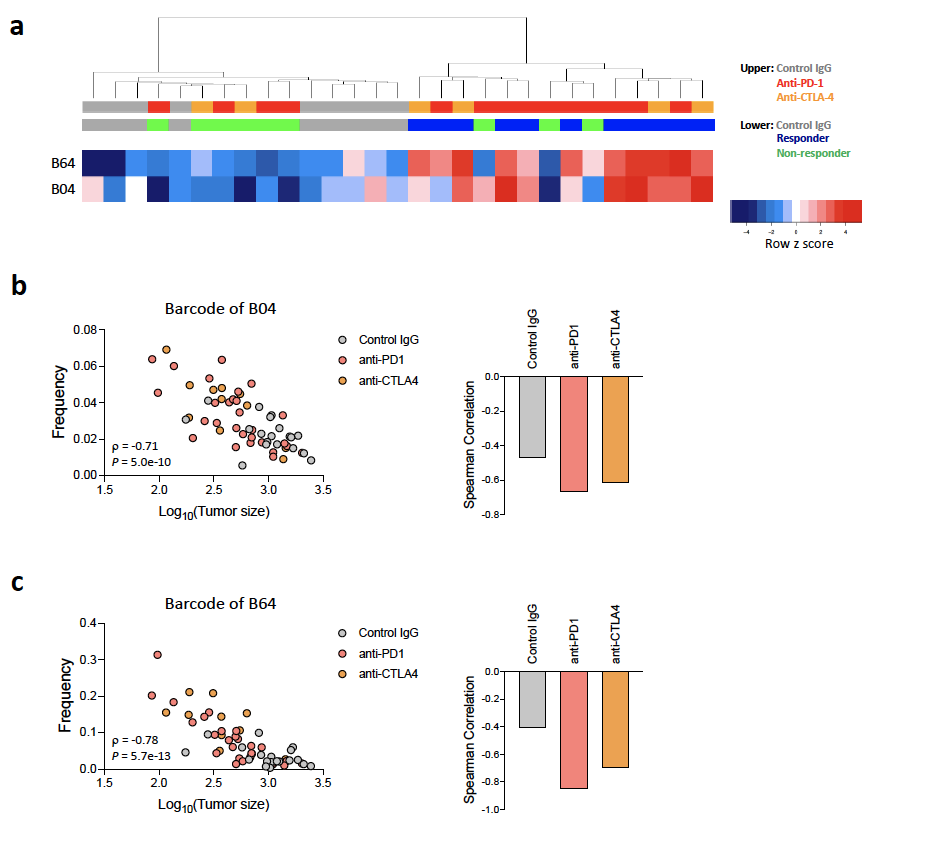


**Fig. S5. Frequency of clones representing line B04 or B64 negatively correlates with tumor size.**

(a) Heatmap of abundance of barcodes representing line B04 or B64 in samples plotted in Fig. 3c. The order of samples is the same as in Fig. 3c. Only information of B04 and B64 is shown. (b-c) Frequency of the barcodes representing (b) line B04 or (c) line B64 in all samples. Their frequencies are negatively correlated with tumor size in each group.


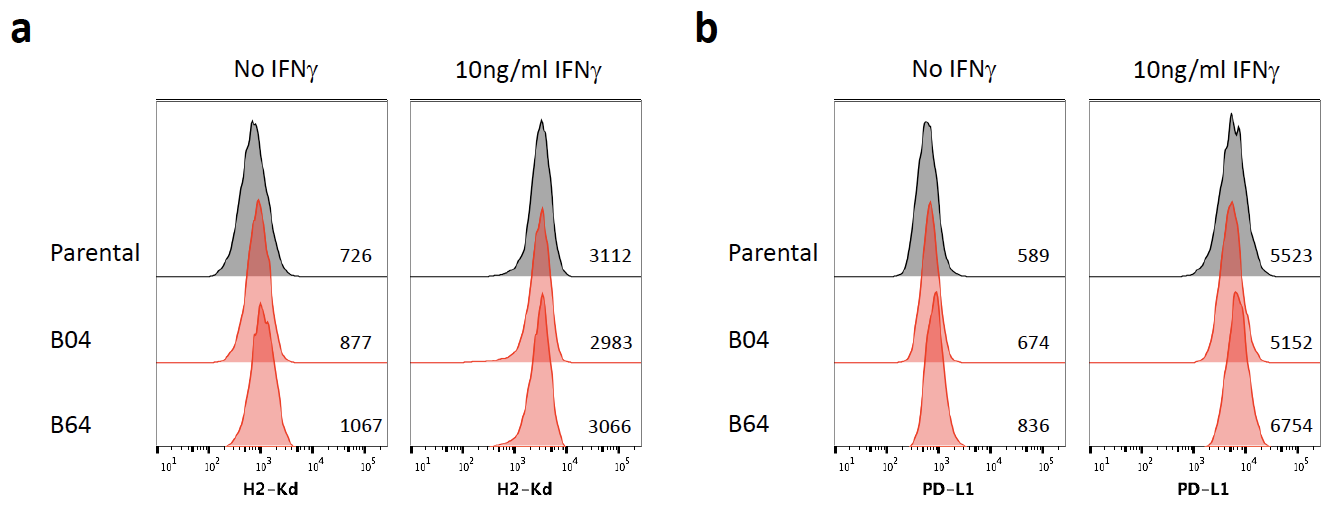


**Fig. S6. ICB-resistant lines have similar response to IFNγ.**

(a-b) Parental CT26 after *in vivo* selection, line B04, or line B64 were treated by vehicle control or 10ng/ml IFNγ for 2 days. Line B04 and B64 exhibit similar induction of (a) MHC-I and (b) PD-L1 in response to IFNγ. Median fluorescence intensities are indicated in each plot.


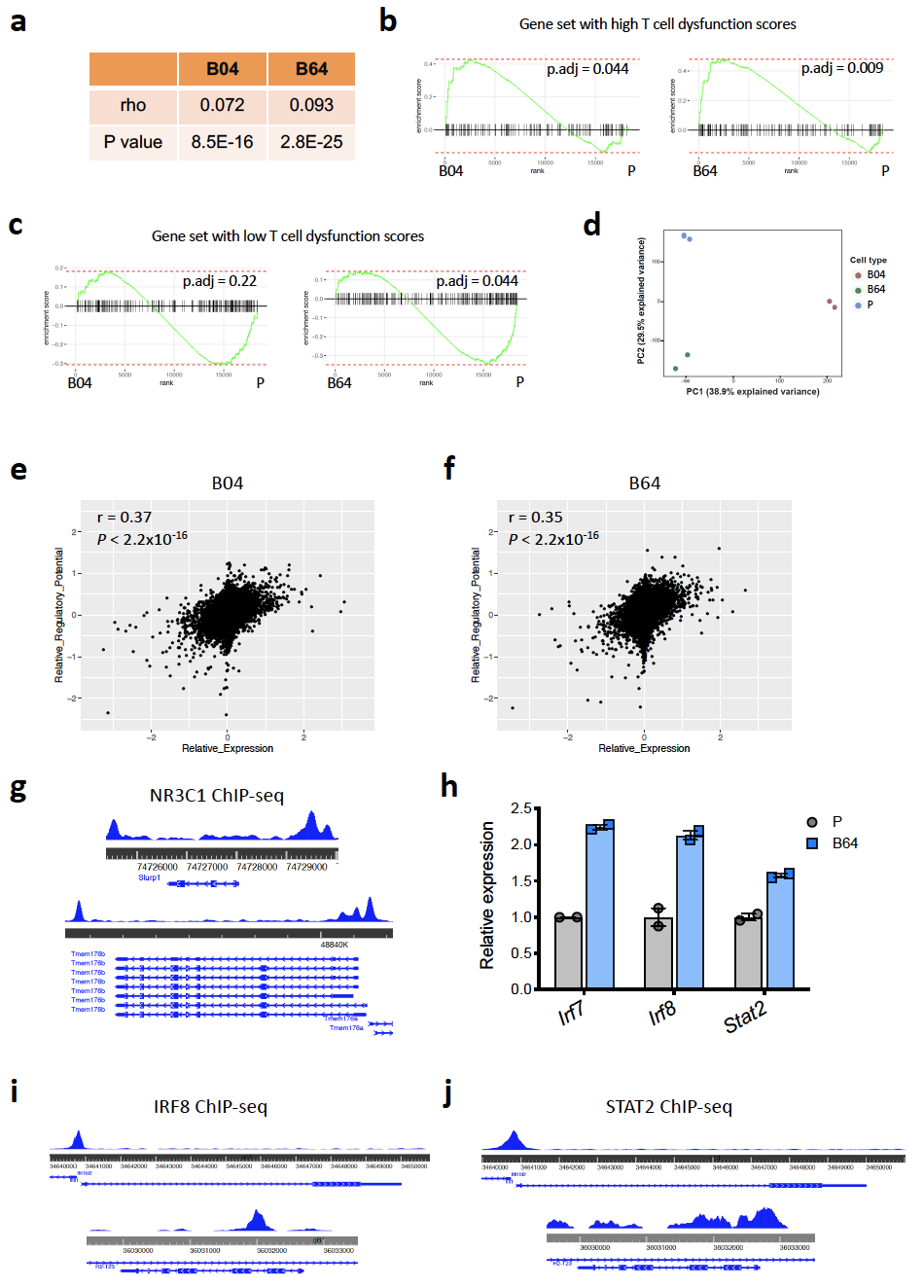


**Fig. S7. Resistant lines show heterogeneous epigenetic profiles.**

(a) Transcriptome of lines B04 and B64 positively correlates with TIDE T cell dysfunction score. (b-c) GSEA analysis of genes with the highest (b) or lowest (c) T cell dysfunction scores, comparing expression between lines B04/B64 and the parental line. (d) PCA of ATAC-seq data from parental line CT26 and ICB-resistant lines B04 and B64 suggests distinct expression profiles between the three lines. (e-f) Gene expression based on RNA-seq positively correlates with gene regulatory potential inferred from ATAC-seq for line B04 (e) and line B64 (f). (g) GR can bind to the promoter and/or enhancer region of *Slurp1* and *Tmem176b* according to published ChIP-seq data [[73]](https://paperpile.com/c/lPEkh9/tNrbJ). (h) *Irf7*, *Irf8*, and *Stat2* are over-expressed in line B64. (i-j) IRF8 (i) or STAT2 (j) can bind to the promoter and/or enhancer region of *Ifit1* and *H2-T23* according to published ChIP-seq data [[106]](https://paperpile.com/c/lPEkh9/YzdId).


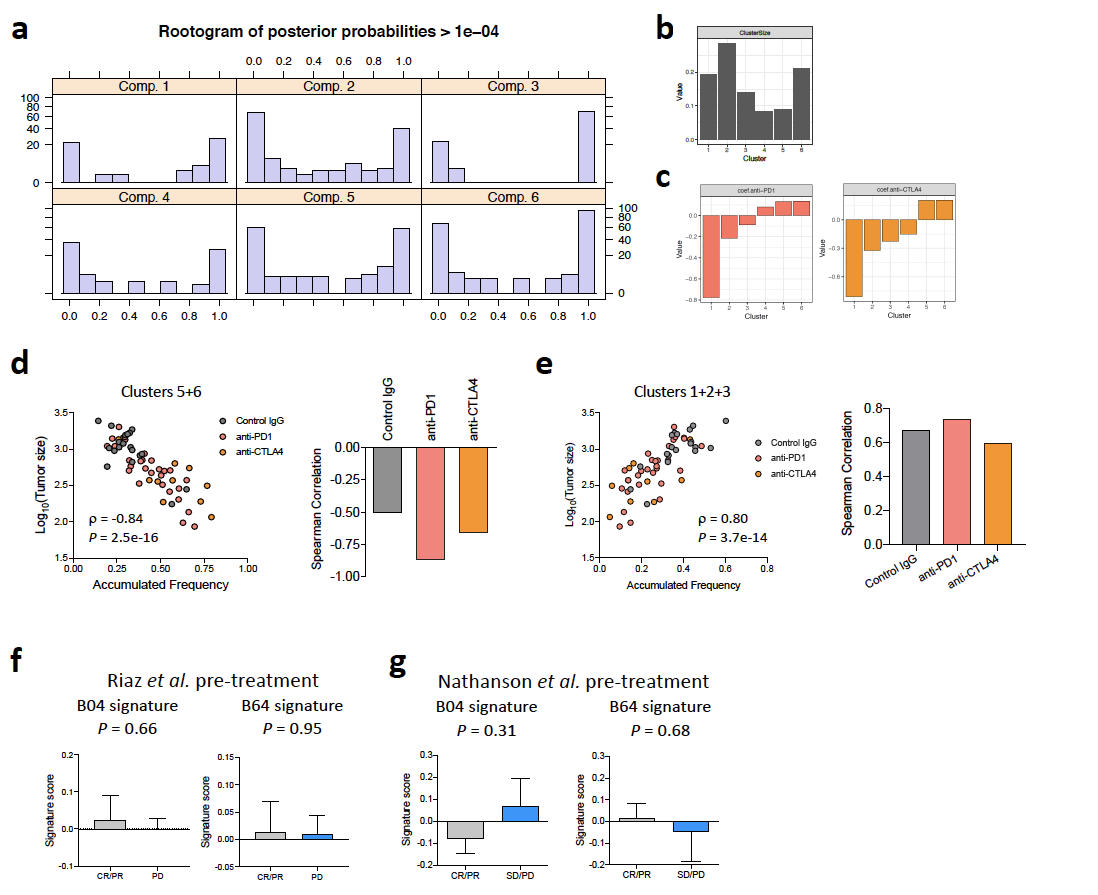


**Fig. S8. Mathematical modeling of tumor growth dynamics infers the contribution of cancer cells to tumor growth under different conditions.**

(a) Rootogram of posterior probabilities of clonal assignment for each cluster, inferred from the Flexmix model. (b) Cluster size of the seven clusters of cancer clones with different growth patterns *in vivo*. (c) Coefficients of anti-PD-1 or anti-CTLA-4 treatment in the model. A positive value indicates that the clone frequency at tumor harvest is higher in the corresponding ICB treatment than the control treatment. (d) The cumulative frequency of clones belonging to cluster 5 or 6 in panel (a) positively correlates with the post-treatment tumor size in all treatment groups. (e) The cumulative frequency of clones belonging to cluster 1, 2, or 3 in panel (a) positively correlates with the post-treatment tumor size in all treatment groups. (f-g) Cancer cell-intrinsic resistance signatures derived from line B04 or B64 shows no significant correlation with ICB response within pre-treatment samples in the (f) Riaz et al. study [[54]](https://paperpile.com/c/lPEkh9/BnghI) and (g) Nathanson et al. study [[84]](https://paperpile.com/c/lPEkh9/4Lwp). (mean ± s.d.; Two-sided t-test)

**References**

106. Mancino A, Termanini A, Barozzi I, Ghisletti S, Ostuni R, Prosperini E, et al. A dual cis-regulatory code links IRF8 to constitutive and inducible gene expression in macrophages. Genes Dev. 2015;29:394–408.
