# Supplementary material for: Clonal tracing reveals diverse patterns of response to immune checkpoint blockade
Source: Genome Biol. 2020 Oct 15;21:263. doi: 10.1186/s13059-020-02166-1 (PMC7559192; doi:10.1186/s13059-020-02166-1)
Supplement: Supplementary file 6 — Additional file 6. Review history. [file 13059_2020_2166_MOESM6_ESM.docx]

Review History

**First round of review**

**Reviewer 1**

**Are you able to assess all statistics in the manuscript, including the appropriateness of statistical tests used?** Yes, and I have assessed the statistics in my report.

**Comments to author:**

The authors report data that a set of proposed biomarkers of response to immune checkpoint blockade (ICB, in this case CTLA4 and PD-1 inhibition) do not stably associate with response across multiple datasets. To evaluate this as a result of potential cell-intrinsic effects, they clonal tracing of CT26 murine tumor evolution in vivo under selection pressure from immune checkpoint blockade. They compared clone frequencies by treatment to assess individual clone effects, showing that tumors responding better to therapy have decreased clonal heterogeneity (consistent with increased selection by an active immune system). Responders had increased frequencies of specific tumor clones that were shown to be resistant to ICB therapy (which was not caused by the known mechanisms of MHC loss or insensitivity to interferon gamma). The authors characterized their ICB resistant clones by RNA-seq and ATAC-seq and derive gene signatures they test for association with ICB response in human trial datasets. In general, the parsing of cell-intrinsic (genetic & epigenetic factors) from cell-extrinsic (immune response, microenvironment, systemic biology) mechanisms of ICB resistance, and understanding how these dynamically interact in any given tumor system, are huge areas of impact for the cancer immunotherapy field.

Comments and Questions:

1. Figure 1a: It's not very surprising that CD274 (PD-L1) RNA expression is not widely associated with clinical response, as the biomarkers in clinical development are based on protein measurements, and PD-L1 RNA expression does not necessarily correlate with protein expression by IHC or flow cytometry. The authors may consider discussing this point.

2. The description of conceptual models in Fig 1D is helpful, however in reality it's likely that both model 1 and model 2 effects obtain in any given patient (which I imagine the authors would agree with). If so, this could be clarified in the discussion as well.

3. Figure 2: Some questions regarding methods: How many animals per group were included in this experiment? Did any of the animals die in the course of this experiment? If so, how were these handled in generating the tumor growth curves (Fig 2b)? Why were only female mice used?

4. Figure 2a: Should "Wide-type" be "Wild-type"?

5. Figure 2b: The error bars by groups in Fig 2b. seem to overlap considerably - Can the authors confirm that the significance values (indicated by " ** " ) here represent results of post-tests from 2-way ANOVA where variables considered are time and treatment group?

6. Figure 2d: It looks like the clonality equation needs the minus and summation symbols inserted in the boxes in the equation editor.

7. Figure 3: The authors claim that clonal constitution is different between responders and non-responders, however was this tested for statistical significance? Why were there more animals in the PD1 and control IgG treated groups than in the CTLA4 treated group?

8. Figure 3: To evaluate these data, it would be helpful to see the individual tumor growth curves for the treatment experiment, stratified by treatment group and classification of responder or non-responder.

9. Figure 3d: It would also be helpful to see statistical comparison of resistance vs non-resistance groups, as well as control IgG vs resistance groups.

10. Figure 3e: The authors should consider including summary data of IHC results, rather than just single replicate example images.

11. Figure 3f: How specifically were the genes selected to include in the heatmap plot? Further, more details need to be included about how the RNA sequencing data analysis was done. For example, the text says "Sequencing reads were mapped to the mm10 genome by RSEM" , however RSEM is not strictly an alignment algorithm (though you can have it call multiple aligners). At a minimum, each algorithm in the bioinformatics workflow should be identified along with the parameter values used (and references to these should be included in the manuscript).

12. Figure 3g: How specifically were these gene sets selected to include in the figure?

13. Figure 3j-k: As the response score is a continuous variable, why is it being dichotomized here and shown as high vs low? Is this just for visualization purposes? It would be helpful to see the results of survival modeling association with the score treated as a continuous variable.

14. Figure 4: Were there any genetic differences in clones that were ICB resistant?

15. Figure S6a: It is difficult to know how to interpret these data. The rho values are quite small. And what does it mean that differentially expressed genes in tumor cell lines are also in a T cell dysfunction signature derived from bulk tumors (and presumably including the T cells that would be measured as dysfunctional)?

16. Figure 5c: "Lowe" should probably be "Lower"

17. Figure 6c: The authors report the result that tumor size is inversely correlated with frequency of ICB resistant clones in the tumor. What is the explanation for this? Is there a dynamic or kinetic rationale based on when the tumor samples were collected? Are these tumors smaller because there is less immune infiltration at the relevant time point? Are non-responding tumors simply under different selection pressures systemically in the microenvironment such that the relative resistance of each clone to T cell killing doesn't matter (i.e. if there is more immune infiltration as the data would suggest, but the immune cells are not able to kill the tumor cells)? Re figure 6d-e, why were signature scores reported as Pearson correlations? Can the authors give additional detail about the methods here? Why was 200 chosen as the number of differentially regulated genes to include in signature generation? Were sensitivity analysis or cross-validation of this number done? What is meant by taking the Pearson correlation between a 200 gene signature and a transcriptome, and what is the rationale for doing this (rather than a simpler approach, such as scoring each tumor with a signature value and comparing these values between responders vs. non-responders)?

18. General points re generalizability: Can the authors comment on the degree to which the resistance signatures they develop based on clonal frequency studies are also applicable in other models and/or human tumors?

**Reviewer 2**

**Are you able to assess all statistics in the manuscript, including the appropriateness of statistical tests used?** Yes, and I have assessed the statistics in my report.

**Comments to author:**

The authors presented an interesting and timely experiment using mouse model system to study clonal selection in immunotherapy. The experiments and bioinformatics analyses were comprehensive. This is overall a nice study to add to the current literature.

The paper is rather densely written and hence difficult to follow. When looking further in details, the reviewer has the following concerns:

1. It is unclear what is the main message of the paper. Finding cancer clones that remained in the responsive tumors to be resistant to treatment is not new. Cancer intrinsic and extrinsic factors work together to determine treatment response is not new.

2. "Taken together, these results indicate that ICB responders and non-responders show different evolutionary trajectories despite sharing the same cancer clones. It also reveals the intrinsic limitation of using genomic mutation information to predict ICB response, and underlies the insights provided from tumor transcriptomics data."

This statement is too strong. Only barcode DNA was sequenced, which identified the initiating cancer clone that was injected into the mouse. As a new tumor grows, evolution of cancer cells happens and new mutations may be acquired in the process. The reviewer did not find anywhere in the paper that mutation information was obtained from the dissected tumors. So this statement needs to be supported by actually looking at the genomic mutation information during the progression of tumors. The reviewer understands the study design of the mouse experiments may not allow for such practice. But then that would be a major limitation of this study, a mis-alignment between mouse experiments and real patient study where tumors exist before treatment, hence mouse experiment specific, and does not "reveal" much biology as stated above. There are a lot of overstatements as such throughout the paper. Another example would be going from enrichment of resistant clones in responsive tumors to stating an enrichment of sensitive clones in non-responsive tumors ("These results revealed a surprising phenomenon that the poor ICB-responding tumors may actually contain a higher percentage of ICB-sensitive cancer cells during ICB treatment), which is negated by the authors' own results in Fig3C where almost all clones seemed to be expressed in non-responders.

2. The definition of responder vs. non-responder was: "tumors larger than 10^(M-0.1) were classified as "non-responder", and tumors smaller than 10^(M-0.5) were classified as "responder" (M denotes the logarithmic median tumor size)". So not all mouse tumors were classified. There were tumors that fall under between the two sizes which would be important to study and compare as well. The majority of the paper was focused on these "extreme" cases, which is interesting but still does not provide a full picture that would be useful for studying ICB response. Towards the end, the authors switched to talking about the full spectrum in Fig6C in that the frequency of the resistant clone is negatively correlated with tumor size. This inconsistency needs to reconciled throughout the paper.

3. "Upon genotyping of the barcode in each line, we identified 5 lines (numbered A01, A06, B04, B64 and C32) representing 2 distinct barcoded clones that were significantly enriched in the ICB responders…" After this statement, only B04 and B64 were studied thoroughly. What was the justification and selection criteria?

4. Any plausible interpretation on the transcriptional differences in B04 and B64?

6. Figure 3C: explain further the total # of barcodes (equal to clones?), which ones are B04, B64. The legend is illegible.

7. Figure 3I: add confidence intervals to HR.

Minor comments:

Fig2d: The equation does not show correctly.

**Authors Response**

**Point-by-point responses to the reviewers’ comments:**

**Response to reviewer’s comments**

Reviewer #1: The authors report data that a set of proposed biomarkers of response to immune checkpoint blockade (ICB, in this case CTLA4 and PD-1 inhibition) do not stably associate with response across multiple datasets. To evaluate this as a result of potential cell-intrinsic effects, they clonal tracing of CT26 murine tumor evolution in vivo under selection pressure from immune checkpoint blockade. They compared clone frequencies by treatment to assess individual clone effects, showing that tumors responding better to therapy have decreased clonal heterogeneity (consistent with increased selection by an active immune system). Responders had increased frequencies of specific tumor clones that were shown to be resistant to ICB therapy (which was not caused by the known mechanisms of MHC loss or insensitivity to interferon gamma). The authors characterized their ICB resistant clones by RNA-seq and ATAC-seq and derive gene signatures they test for association with ICB response in human trial datasets. In general, the parsing of cell-intrinsic (genetic & epigenetic factors) from cell-extrinsic (immune response, microenvironment, systemic biology) mechanisms of ICB resistance, and understanding how these dynamically interact in any given tumor system, are huge areas of impact for the cancer immunotherapy field.

Comments and Questions:
1. Figure 1a: It's not very surprising that CD274 (PD-L1) RNA expression is not widely associated with clinical response, as the biomarkers in clinical development are based on protein measurements, and PD-L1 RNA expression does not necessarily correlate with protein expression by IHC or flow cytometry. The authors may consider discussing this point.

We agree that the difference between RNA transcription and protein level is an important point. The observation that PD-L1 RNA level is not always associated with anti-PD-1/anti-PD-L1 response can be due to poor predictive power of PD-L1 per se or poor correlation of RNA and protein expression for PD-L1. We searched the literature for studies that used PD-L1 protein expression level as a biomarker for response, and found that the prediction power varies even when using protein levels (Le et al., NEJM, 2015; Llosa et al., Cancer Immunol Res, 2019). We added this to the Results section in the revision.

2. The description of conceptual models in Fig 1D is helpful, however in reality it's likely that both model 1 and model 2 effects obtain in any given patient (which I imagine the authors would agree with). If so, this could be clarified in the discussion as well.

We agree that both cancer-cell-intrinsic and cancer-cell-extrinsic factors may influence ICB response in any given patient. We have added this clarification to the first section of Results and to the Discussion.

3. Figure 2: Some questions regarding methods: How many animals per group were included in this experiment? Did any of the animals die in the course of this experiment? If so, how were these handled in generating the tumor growth curves (Fig 2b)? Why were only female mice used?

There were 10 mice in the control IgG group, 15 mice in the anti-PD-1 group, and 10 mice in the anti-CTLA-4 group, with 2 implantation sites per mouse. Previous studies (Kim et al., PNAS, 2014) indicated that the response to anti-PD-1 in this model was heterogeneous, so we included more mice in the anti-PD-1 group to improve the statistical power to detect a difference in tumor growth, and also to harvest enough tumors in each response category. On day 22, we sacrificed all mice for analyses. No mice died before this time point. Gender can be a confounding factor for immune response, as reported in a recent study (Ye et al., Nat Comm, 2020). To improve the consistency in baseline tumor growth across different recipients and optimize signal-to-noise ratio, we used mice of the same gender to control for this confounding factor. Most published papers use either males or females, not mixed recipients (e.g. Zhang et al., Nature, 2018 used all female recipients; Pan et al., Science, 2018 used all male recipients; Burr et al., Cancer Cell, 2019 used all female recipients). We now included this information in the Figure Legend in the revision.

4. Figure 2a: Should "Wide-type" be "Wild-type"?

We thank the reviewer for catching this typo and have corrected it in the revised manuscript.

5. Figure 2b: The error bars by groups in Fig 2b. seem to overlap considerably - Can the authors confirm that the significance values (indicated by " ** " ) here represent results of post-tests from 2-way ANOVA where variables considered are time and treatment group?

We plotted the error bar as standard deviation (s.d.) in this figure, which is why there is overlap. If we plot the error bar as standard error of the mean (or s.e.m., which equals to s.d. divided by n-1 and is the parameter used in statistical tests to calculate p value), the error bars show better separation, as shown below.

We have confirmed that the significance values shown in Figure 2b accurately represent the 2-way ANOVA post-test results.

6. Figure 2d: It looks like the clonality equation needs the minus and summation symbols inserted in the boxes in the equation editor.

We corrected this in the revision.

7. Figure 3: The authors claim that clonal constitution is different between responders and non-responders, however was this tested for statistical significance? Why were there more animals in the PD1 and control IgG treated groups than in the CTLA4 treated group?

We performed the Kolmogorov-Smirnov test to examine the differences in distribution of barcode frequencies. The responder group shows a substantially different distribution compared to the non-responder and control IgG groups. We now include this information in the revision (Fig. S3c).

In addition, in Fig. 3d, the clonality of distribution is significantly higher in responders than non-responders.

We also tested the frequencies of individual barcodes between responders and non-responders. Over 100 barcodes showed differential frequencies, even after multiple comparison adjustment of *P* value.

As described in #3 above, previous studies indicated that the response to anti-PD-1 in CT26 was heterogeneous, so we included more mice in the anti-PD-1 group to improve the statistical power to detect a difference in tumor growth, and also to harvest enough tumors in each response category. Anti-CTLA-4 was known to have a strong therapeutic effect in this model, so non-responders are rare. Several responders showed complete response, leaving no detectable residual tumor to assay for barcode distribution and clonality.

8. Figure 3: To evaluate these data, it would be helpful to see the individual tumor growth curves for the treatment experiment, stratified by treatment group and classification of responder or non-responder.

We thank the reviewer for this suggestion and have added it Fig. S3a in the revision, with responders marked in blue and non-responders marked in green.

9. Figure 3d: It would also be helpful to see statistical comparison of resistance vs non-resistance groups, as well as control IgG vs resistance groups.

We added the suggested comparisons to our analyses and updated the figure in the revision. The clonality in responders significantly differ from that of non-responders, while the clonality in the control IgG group is not significantly different from the non-responders.

10. Figure 3e: The authors should consider including summary data of IHC results, rather than just single replicate example images.

We thank the reviewer for this suggestion and have added the summary data of IHC results to Fig. 3e and moved the representative images to Fig. S3.

11. Figure 3f: How specifically were the genes selected to include in the heatmap plot? Further, more details need to be included about how the RNA sequencing data analysis was done. For example, the text says "Sequencing reads were mapped to the mm10 genome by RSEM", however RSEM is not strictly an alignment algorithm (though you can have it call multiple aligners). At a minimum, each algorithm in the bioinformatics workflow should be identified along with the parameter values used (and references to these should be included in the manuscript).

We thank the reviewer for pointing out this confusing description in our original manuscript, and have provided clarified description in the manuscript.

Gene selection for heatmap plot: We first performed gene set enrichment analysis of the differential gene expression between responders and non-responders. Within the gene sets that were enriched, we picked genes with highly significant log_2_-fold-change to plot in the heatmap.

Analysis of RNA-seq data: RNA-seq fastq reads were aligned to the mm10 genome using STAR v2.5 (Dobin et al., Bioinformatics, 2013) and quantified using RSEM (Li et al., BMC Bioinformatics, 2011). The downstream differential gene expression analysis was performed using DESeq2 (Love et al., Genome Biol, 2014) and differentially regulated gene sets were derived by GSEA (Subramanian et al., PNAS, 2005). All the analyses used default paramter settings.

We switched the order of Fig. 3f and 3g in the revision and added these explanations to the Figure Legend and the Methods section of the revised manuscript.

12. Figure 3g: How specifically were these gene sets selected to include in the figure?

We selected top enriched gene sets that reflect different aspects of biological processes to include in the figure. The majority of top enriched gene sets are related to T-cell or B-cell-mediated immunity. We therefore included one gene set for each cell type to show in the Figure. We also include the entire list of enriched gene sets along with their FDRs in the Supplementary Table 2 of the revised manuscript, and refer to Table S2 in the Figure Legend.

13. Figure 3j-k: As the response score is a continuous variable, why is it being dichotomized here and shown as high vs low? Is this just for visualization purposes? It would be helpful to see the results of survival modeling association with the score treated as a continuous variable.

We thank the reviewer for this great suggestion. In the revision, we still retained the dichotomized survival curves for visualization purposes, but now show p-values from a Cox-PH regression treating the signature as a continuous variable. In addition, we changed Fig. 3l from showing hazard ratio to showing the Cox-PH regression coefficient of the signature score.

14. Figure 4: Were there any genetic differences in clones that were ICB resistant?

We performed VarScan analysis of RNA-seq data from parental CT26 line, line B04, and line B64 to call genetic differences against the reference mm10 genome. For B04, we found an Arhgap30 (Rho GTPase Activating Protein 30) L458S mutant not detected in the parental line or B64. Literature research revealed that this mutant is likely a germline SNP present in Balb/c background lines, and has no known impact on immune function. Arhgap30 gene expression is not detectable in the parental line or line B64, which is why the mutation was detected only in the RNA-seq of B04. For B64, we did not detect any unique genetic mutation. These results suggest epigenetic mechanisms of resistance in line B04 and B64. We have added this description in the revised manuscript.

15. Figure S6a: It is difficult to know how to interpret these data. The rho values are quite small. And what does it mean that differentially expressed genes in tumor cell lines are also in a T cell dysfunction signature derived from bulk tumors (and presumably including the T cells that would be measured as dysfunctional)?

In our previous TIDE study (Jiang et al., Nat Med, 2018), we performed Cox-PH regression to identify genes whose expression in the bulk tumor is associated with compromised anti-tumor activity of T cells (or “T cell dysfunction”). Based on the regression, we calculated a z score for each gene and used the z scores for all genes to derive a T-cell dysfunction signature. Samples with high expression of genes with positive z score and low expression of genes with negative z score are expected to show T cell dysfunction.

These genes included those expressed in cancer cells and/or those expressed in immune cells. We expected that some cancer-cell-intrinsic immune resistance genes may also be identified to be correlated with T cell dysfunction in TIDE analysis, and therefore performed a correlation assay of the two signatures. Indeed, we found positive correlation. Because it is genome-wide level correlation, the correlation coefficient is usually very small (typically between -0.1 and 0.1, as was also shown in Fig. 3a, 3c, and 3d from the original TIDE paper). We realize that this might be difficult to interpret for the general readers, and therefore added a GSEA analysis to complement Fig. S7.

Specifically, we focused on genes with high or low z scores for T-cell dysfunction from the TIDE study. Genes with strong positive TIDE dysfunction z score, whose higher expression indicates cytotoxic T-cell dysfunction, were up-regulated in the ICB-resistant lines, whereas those with negative TIDE z score were unchanged or down-regulated.

16. Figure 5c: "Lowe" should probably be "Lower"

We thank the reviewer for catching this typo and have corrected it in the revised manuscript.

17. Figure 6c: The authors report the result that tumor size is inversely correlated with frequency of ICB resistant clones in the tumor. What is the explanation for this?

The clonal frequencies were obtained after ICB treatment and reflect the degree of immune selection. We think this is a very interesting observation and can be explained by conditional clonal selection of cancer cells by the immune system. Specifically, for tumors grown in different recipient mice, there is inter-mouse heterogeneity in intratumoral immune infiltration, which can be caused by the differences in individual immune health or the stochasticity of TCR/BCR sequences in each individual mouse. For tumors that happen to have higher T cell infiltration, their size tends to be smaller, likely due to anti-cancer immunity. In these tumors, the ICB-resistant clones also tend to be enriched, potentially due to strong immune selection to reduce the vast majority of other clones. Therefore, tumor size is inversely correlated with frequency of ICB resistant clones in the tumor.

17a. Is there a dynamic or kinetic rationale based on when the tumor samples were collected? Are these tumors smaller because there is less immune infiltration at the relevant time point?

We collected the post-treatment tumor samples on Day 22 post-transplantation for DNA/RNA sequencing. The time for sample collection follows traditional experiments in CT26 (Kim et al., PNAS, 2014), which ensures sufficient material collection to evaluate both ICB response and barcode distribution. The smaller tumors had higher immune cell infiltration at the time of tumor harvest (post ICB treatment). As described above, the most likely explanation is that the anti-tumor immunity of the tumor-infiltration immune cells led to smaller tumor size.

17b. Are non-responding tumors simply under different selection pressures systemically in the microenvironment such that the relative resistance of each clone to T cell killing doesn't matter (i.e. if there is more immune infiltration as the data would suggest, but the immune cells are not able to kill the tumor cells)?

We agree with the reviewer that non-responding tumors were under different selection pressures in the hosts, despite our efforts to use mice of the same age / gender / diet. This, in turn, influences T-cell infiltration in the microenvironment. We concur with the reviewer’s hypothesis that in these non-responding tumors, the relative resistance of each cancer clone to T-cell-driven killing might not matter.

17c. Re figure 6d-e, why were signature scores reported as Pearson correlations? Can the authors give additional detail about the methods here?

The signature scores were calculated as the Pearson correlation between bulk tumor gene expression and line B04/B64 gene expression. The method was published in our previous TIDE paper (Jiang et al., Nat Med, 2018), which worked well in assessing a clinical sample’s T-cell-exhaustion or T-cell-exclusion signature. We provide the detailed explanation of the method as follows. We first defined B04 (or B64) gene signature as the log_2_-fold-change of genes in each line compared to the parental line. For each tumor within one clinical cohort (e.g. the Riaz pre-treatment cohort), we normalized log_2_-TPM of gene *i* in sample j as the deviation from the mean log_2_-TPM of gene *i* in all samples in the cohort:

Ei,j'=Ei,j-Ei

To derive the B04 (or B64) signature score in a tumor sample, we calculated the Pearson correlation between the reference signature (the log_2_-fold-change from the first step) and the normalized gene expression in the tumor sample (Ei,j'). If a sample has high expression of genes that are also highly expressed in line B04 (or B64), and low expression of genes that are also lowly expressed in line B04 (or B64), then this sample should have a high B04 (or B64) signature score. We used the most up/down-regulated genes in B04 and B64 for signature score calculation in order to focus on the genes that define the difference between these lines and the parental line and eliminate the noise that can be introduced by the genes with low expression variance. Similar approaches have been used for many purposes, such as classification (e.g. choosing the most variable genes for unsupervised clustering) or scRNA-seq analysis (e.g. choosing the top principle components for single cell clustering).

17d. Why was 200 chosen as the number of differentially regulated genes to include in signature generation? Were sensitivity analysis or cross-validation of this number done?

We performed robustness test (e.g. using the top 200, 400, 800, or all significantly differentially expressed genes between line B04/B64 and the parental line) and found the conclusion to be robust under different parameters. Shown below is the result by using all significantly differentially expressed genes to calculate signature score. Cancer-cell-intrinsic signatures of B04 and B64 were enriched in ICB responders in on-treatment/post-treatment samples, but not pre-treatment samples:

17e. What is meant by taking the Pearson correlation between a 200 gene signature and a transcriptome, and what is the rationale for doing this (rather than a simpler approach, such as scoring each tumor with a signature value and comparing these values between responders vs. non-responders)?

We provided more detailed answer to this reviewer’s earlier question (17c). To avoid confusion, we have changed the label on the Y axis to “Signature score”, instead of “Pearson correlation”. We have added a detailed description in the Methods section.

18. General points re generalizability: Can the authors comment on the degree to which the resistance signatures they develop based on clonal frequency studies are also applicable in other models and/or human tumors?

We tested the clonal-specific signatures of B04 and B64 in multiple ICB treatment cohorts (Fig. 6d-g, Fig. S8f-g). In the two ICB cohorts (both melanoma) where on-treatment/post-treatment samples are available, the cancer-cell-intrinsic signature scores of these samples matched the results of our mouse study. In addition, we found positive correlation between T cell infiltration levels and the cancer-cell-intrinsic resistance signature in multiple other cancer cohorts where only pre-treatment samples are available (such as bladder cancer and kidney cancer), consistent with the model that higher T cell infiltration selects for cancer cells with stronger immune resistance. Therefore, we expect that the clonal resistance signatures to be applicable to other cancer types, which can be tested in future studies with both pre-treatment and on-treatment/post-treatment samples’ RNA-seq data. It is important to note though, that the two resistant lines we isolated have intact antigen presentation capacity, allowing these cells to be recognized by the T cells. Antigen presentation defect, such as by genetic deletion or epigenetic silencing, is known to be a major mechanism of cancer-cell-intrinsic resistance to ICB (Garrido et al., Curr Opin Immunol, 2016). Therefore our findings present alternative resistance signatures independent of antigen presentation defects.

Reviewer #2: The authors presented an interesting and timely experiment using mouse model system to study clonal selection in immunotherapy. The experiments and bioinformatics analyses were comprehensive. This is overall a nice study to add to the current literature.

The paper is rather densely written and hence difficult to follow. When looking further in details, the reviewer has the following concerns:

1. It is unclear what is the main message of the paper. Finding cancer clones that remained in the responsive tumors to be resistant to treatment is not new. Cancer intrinsic and extrinsic factors work together to determine treatment response is not new.

We thank the reviewer for this question, which helped us improve our writing and more clearly emphasize the main findings and novelty of this study.

The reviewer is correct - the concepts of immunoediting and intrinsic/extrinsic factors regulating anti-tumor immunity are not new. The novelty of this study is the observation that that even for identical labeled cancer cells grown in isogenic individuals, the immune response can be diverse. This finding has two important implications for clinical treatment and translational research.

First, our results indicate that there is an inherent limitation in using pre-treatment genetic information to guide immunotherapy treatment decisions, because tumors with a defined genetic background can still respond differently. Therefore, longitudinal data on the tumor microenvironment, such as by RNA-seq of biopsies, will likely help optimize treatment strategies.

Second, our study reveals a paradoxical observation that may affect interpretations of tumor RNA-seq data for response-signature association studies. When studying biomarkers for ICB response/resistance, it is common to use RNA-seq from tumors that respond to ICB as the “responder” signature. Our study suggests that it is possible that immune-resistant cancer cells are enriched in such samples, thus the “responder” signature derived this way might be enriched for cancer-cell-intrinsic resistance signature. This might also contribute to the variable performance of ICB response biomarkers in different clinical cohorts.

We have clarified these points in our revised manuscript in the Discussion.

2. "Taken together, these results indicate that ICB responders and non-responders show different evolutionary trajectories despite sharing the same cancer clones. It also reveals the intrinsic limitation of using genomic mutation information to predict ICB response, and underlies the insights provided from tumor transcriptomics data."

This statement is too strong. Only barcode DNA was sequenced, which identified the initiating cancer clone that was injected into the mouse. As a new tumor grows, evolution of cancer cells happens and new mutations may be acquired in the process. The reviewer did not find anywhere in the paper that mutation information was obtained from the dissected tumors. So this statement needs to be supported by actually looking at the genomic mutation information during the progression of tumors. The reviewer understands the study design of the mouse experiments may not allow for such practice. But then that would be a major limitation of this study, a mis-alignment between mouse experiments and real patient study where tumors exist before treatment, hence mouse experiment specific, and does not "reveal" much biology as stated above.

We agree with the reviewer that accumulation of new mutations is an important aspect of tumor evolution in human cancers, which is poorly simulated in existing mouse models. This is an inherent limitation of using mouse models to study human cancer, where the experiments are conducted in a matter of a few weeks. To avoid confusion, we have changed the term “evolutionary trajectory” to “response pattern” or “clonal constitution”. We also now point out this critical difference between mouse models and human tumors in the discussion.

Nonetheless, our study reveals diverse response patterns of tumors that initiated from the same cell line. It indicates that even if the genetic background of cancer is defined prior to ICB treatment, there can be diverse response patterns, which are correlated with intra-tumoral immune infiltration and might be attributable to each individual’s specific immune health. Our study does not downplay the importance of genomic mutation profiling, which is critical for precision medicine and immunotherapy. Instead, we propose the importance of transcriptional data as a complementary source of information to guide immunotherapy.

There are a lot of overstatements as such throughout the paper. Another example would be going from enrichment of resistant clones in responsive tumors to stating an enrichment of sensitive clones in non-responsive tumors ("These results revealed a surprising phenomenon that the poor ICB-responding tumors may actually contain a higher percentage of ICB-sensitive cancer cells during ICB treatment), which is negated by the authors' own results in Fig3C where almost all clones seemed to be expressed in non-responders.

We wish to clarify that the statement referenced above does not conflict with our results in Fig. 3c. We plotted the accumulative abundance of these putative ICB-sensitive clones in responders and non-responders in the revised manuscript (updated Fig. 6), with the same criteria for responder/non-responder as in Fig. 3. Indeed, these clones showed higher cumulative percentage in non-responders than responders. It is important to note that these are post-treatment samples. A plausible explanation for this observation is that the responders had overall better systemic immune health, thus allowing better T cell infiltration and enriching for ICB-resistant cancer clones during/after ICB treatment. This would consequently cause ICB responders to harbor a higher percentage of ICB-resistant clones and a lower percentage of ICB-sensitive clones than the non-responders.

To avoid confusion, we clarified this statement as “These results revealed that the non-responders may actually contain a higher percentage of ICB-sensitive cancer cells post ICB treatment”. We also added the frequencies of these clones in responders and non-responders to the revised manuscript.

3. The definition of responder vs. non-responder was: "tumors larger than 10^(M-0.1) were classified as "non-responder", and tumors smaller than 10^(M-0.5) were classified as "responder" (M denotes the logarithmic median tumor size)". So not all mouse tumors were classified. There were tumors that fall under between the two sizes which would be important to study and compare as well. The majority of the paper was focused on these "extreme" cases, which is interesting but still does not provide a full picture that would be useful for studying ICB response. Towards the end, the authors switched to talking about the full spectrum in Fig6C in that the frequency of the resistant clone is negatively correlated with tumor size. This inconsistency needs to reconciled throughout the paper.

We thank the reviewer for raising this point, and reconciled this in the revision by adding the analysis of all tumors in the earlier figures and adding the analysis on responder/non-responders for results presented in Fig. 6c.

Specifically, in Fig. 3d, we compared barcode clonality in responders and non-responders. We added clonality for all tumors to Fig. S3 in the revision. In Fig. 4b, we compared the percentages of B04 and B64 in responders and non-responders. We added the percentages of these clones in all tumors to Fig. S5 in the revision. In Fig. 6c we assessed the cumulative frequency of resistant clones in all tumors. We added the comparison of this frequency between responders and non-responders to the updated Fig. 6c and moved the comparison for all tumors to Fig. S8. We show the updated analyses as below. In summary, we found that ICB-responders exhibited stronger clonal selection for ICB-resistant clones.

4. "Upon genotyping of the barcode in each line, we identified 5 lines (numbered A01, A06, B04, B64 and C32) representing 2 distinct barcoded clones that were significantly enriched in the ICB responders…" After this statement, only B04 and B64 were studied thoroughly. What was the justification and selection criteria?

The justification is that A01, A06, and B04 share the same barcode and thus share the same ancestor clone. Likewise, B64 and C32 share the same barcode from ancestor clone. We picked one line from each clonal group for downstream mechanistic study, reasoning that the different clonal groups may harbor different resistance mechanisms.

5. Any plausible interpretation on the transcriptional differences in B04 and B64?

Based on the barcode identity, B04 and B64 come from two different ancestor clones, so different transcriptomes are not unexpected.

RNA-seq showed that compared to the parental line, B04 has elevated expression of genes in DNA replication and sterol metabolism, and B64 has elevated interferon response gene expression. Several differentially expressed genes, including *Slurp1* and *Tmem176b* up-regulated in line B04 and *Ifit1* and *H2-T23* up-regulated in line B64, have been reported to show immunosuppressive effects.

Regarding what mechanisms may underlie the differential transcription for B04 and B64, we looked into the mutation information extrapolated from RNA-seq. We found *Arhgap30* (Rho GTPase Activating Protein 30) L458S mutation in B04 but absent in parental line and B64, and did not find a B64-specific mutation. *Arhgap30* was expressed in B04 but not detectable in parental line or B64, which might be why we detected this SNP only in B04. Although this gene was reported to be related to be correlated with LDL cholesterol levels, we do not know what underlies the differential expression of this gene. We therefore speculate that the transcriptional difference might be driven by epigenetic factors. We have added this description in the revised manuscript.

6. Figure 3C: explain further the total # of barcodes (equal to clones?), which ones are B04, B64. The legend is illegible.

Yes, we used “barcodes” and “clones” interchangeably in the original manuscript. To avoid confusion, we replaced “clone” with “barcode” in the revision. We selected the union of most abundant barcodes in each sample (the top 90% of barcode reads in each sample, 839 barcodes in total) to plot this heatmap. In the revised manuscript, we reordered the rows of the heatmap, ranked by the difference between the responders and the control IgG group. Because rows are packed very dense, it is hard to clearly see the specific row for B04 and B64. We mark these clones as below and include the heatmap for just B04 and B64 in Fig. S5 in the revised manuscript. We updated the legend to explain the upper and lower color bars in the revision.

7. Figure 3I: add confidence intervals to HR.

For this figure, following Reviewer 1’s suggestion, we switched to showing the results of Cox-PH regression, which treated the signature score as a continuous variable. In the revised manuscript, we show the Cox-PH coefficient value with its standard error, and also the z score.

Minor comments:

Fig2d: The equation does not show correctly.

We have updated the manuscript with the equation correctly shown.

**References**

Burr, M.L., Sparbier, C.E., Chan, K.L., Chan, Y.-C., Kersbergen, A., Lam, E.Y.N., Azidis-Yates, E., Vassiliadis, D., Bell, C.C., Gilan, O., et al. (2019). An Evolutionarily Conserved Function of Polycomb Silences the MHC Class I Antigen Presentation Pathway and Enables Immune Evasion in Cancer. Cancer Cell 36, 385–401.e8.

Dobin, A., Davis, C.A., Schlesinger, F., Drenkow, J., Zaleski, C., Jha, S., Batut, P., Chaisson, M., and Gingeras, T.R. (2013). STAR: ultrafast universal RNA-seq aligner. Bioinformatics 29, 15–21.

Garrido, F., Aptsiauri, N., Doorduijn, E.M., Garcia Lora, A.M., and van Hall, T. (2016). The urgent need to recover MHC class I in cancers for effective immunotherapy. Curr. Opin. Immunol. 39, 44–51.

Jiang, P., Gu, S., Pan, D., Fu, J., Sahu, A., Hu, X., Li, Z., Traugh, N., Bu, X., Li, B., et al. (2018). Signatures of T cell dysfunction and exclusion predict cancer immunotherapy response. Nat. Med. 24, 1550–1558.

Kim, K., Skora, A.D., Li, Z., Liu, Q., Tam, A.J., Blosser, R.L., Diaz, L.A., Jr, Papadopoulos, N., Kinzler, K.W., Vogelstein, B., et al. (2014). Eradication of metastatic mouse cancers resistant to immune checkpoint blockade by suppression of myeloid-derived cells. Proc. Natl. Acad. Sci. U. S. A. 111, 11774–11779.

Le, D.T., Uram, J.N., Wang, H., Bartlett, B.R., Kemberling, H., Eyring, A.D., Skora, A.D., Luber, B.S., Azad, N.S., Laheru, D., et al. (2015). PD-1 Blockade in Tumors with Mismatch-Repair Deficiency. N. Engl. J. Med. 372, 2509–2520.

Li, B., and Dewey, C.N. (2011). RSEM: accurate transcript quantification from RNA-Seq data with or without a reference genome. BMC Bioinformatics 12, 323.

Llosa, N.J., Luber, B., Siegel, N., Awan, A.H., Oke, T., Zhu, Q., Bartlett, B.R., Aulakh, L.K., Thompson, E.D., Jaffee, E.M., et al. (2019). Immunopathologic Stratification of Colorectal Cancer for Checkpoint Blockade Immunotherapy. Cancer Immunol Res 7, 1574–1579.

Love, M.I., Huber, W., and Anders, S. (2014). Moderated estimation of fold change and dispersion for RNA-seq data with DESeq2. Genome Biol. 15, 550.

Pan, D., Kobayashi, A., Jiang, P., Ferrari de Andrade, L., Tay, R.E., Luoma, A.M., Tsoucas, D., Qiu, X., Lim, K., Rao, P., et al. (2018). A major chromatin regulator determines resistance of tumor cells to T cell-mediated killing. Science 359, 770–775.

Subramanian, A., Tamayo, P., Mootha, V.K., Mukherjee, S., Ebert, B.L., Gillette, M.A., Paulovich, A., Pomeroy, S.L., Golub, T.R., Lander, E.S., et al. (2005). Gene set enrichment analysis: a knowledge-based approach for interpreting genome-wide expression profiles. Proc. Natl. Acad. Sci. U. S. A. 102, 15545–15550.

Ye, Y., Jing, Y., Li, L., Mills, G.B., Diao, L., Liu, H., and Han, L. (2020). Sex-associated molecular differences for cancer immunotherapy. Nat. Commun. 11, 1779.

Zhang, J., Bu, X., Wang, H., Zhu, Y., Geng, Y., Nihira, N.T., Tan, Y., Ci, Y., Wu, F., Dai, X., et al. (2018). Cyclin D-CDK4 kinase destabilizes PD-L1 via cullin 3-SPOP to control cancer immune surveillance. Nature 553, 91–95.

**Second round of review**

**Reviewer 1**

The authors have addressed all of my technical concerns and have substantially improved the manuscript.

**Reviewer 2**

The authors have addressed all of my concerns. I have no further comments.
